# Dual-Level Regulation of ACC Synthase Activity by MPK3/MPK6 Cascade and Its Downstream WRKY Transcription Factor during Ethylene Induction in Arabidopsis

**DOI:** 10.1371/journal.pgen.1002767

**Published:** 2012-06-28

**Authors:** Guojing Li, Xiangzong Meng, Ruigang Wang, Guohong Mao, Ling Han, Yidong Liu, Shuqun Zhang

**Affiliations:** 1 College of Life Sciences, Inner Mongolia Agricultural University, Hohhot, Inner Mongolia, China; 2 Division of Biochemistry, Interdisciplinary Plant Group, and Bond Life Sciences Center, University of Missouri, Columbia, Missouri, United States of America; The University of North Carolina at Chapel Hill, United States of America

## Abstract

Plants under pathogen attack produce high levels of ethylene, which plays important roles in plant immunity. Previously, we reported the involvement of ACS2 and ACS6, two Type I ACS isoforms, in *Botrytis cinerea*–induced ethylene biosynthesis and their regulation at the protein stability level by MPK3 and MPK6, two Arabidopsis pathogen-responsive mitogen-activated protein kinases (MAPKs). The residual ethylene induction in the *acs2/acs6* double mutant suggests the involvement of additional ACS isoforms. It is also known that a subset of *ACS* genes, including *ACS6*, is transcriptionally induced in plants under stress or pathogen attack. However, the importance of *ACS* gene activation and the regulatory mechanism(s) are not clear. In this report, we demonstrate using genetic analysis that ACS7 and ACS11, two Type III ACS isoforms, and ACS8, a Type II ACS isoform, also contribute to the *B. cinerea*–induced ethylene production. In addition to post-translational regulation, transcriptional activation of the *ACS* genes also plays a critical role in sustaining high levels of ethylene induction. Interestingly, MPK3 and MPK6 not only control the stability of ACS2 and ACS6 proteins via direct protein phosphorylation but also regulate the expression of *ACS2* and *ACS6* genes. WRKY33, another MPK3/MPK6 substrate, is involved in the MPK3/MPK6-induced *ACS2/ACS6* gene expression based on genetic analyses. Furthermore, chromatin-immunoprecipitation assay reveals the direct binding of WRKY33 to the W-boxes in the promoters of *ACS2* and *ACS6* genes *in vivo*, suggesting that WRKY33 is directly involved in the activation of *ACS2* and *ACS6* expression downstream of MPK3/MPK6 cascade in response to pathogen invasion. Regulation of ACS activity by MPK3/MPK6 at both transcriptional and protein stability levels plays a key role in determining the kinetics and magnitude of ethylene induction.

## Author Summary

Plant immunity, similar to that in animals, also involves mitogen-activated protein kinase (MAPK) cascades. However, plants use unique MAPK substrates and secondary signaling molecules in the process. Among them, ethylene, a gaseous plant hormone, plays critical roles. Ethylene-regulated responses begin with the induction of ethylene biosynthesis. 1-amino-cyclopropane-1-carboxylic acid synthase (ACS) catalyzes the committing and rate-limiting step in ethylene biosynthetic pathway. The Arabidopsis genome encodes nine different ACS isoforms. Two of them, ACS2 and ACS6, were previously shown to be phosphorylated and stabilized by MPK3 and MPK6, two Arabidopsis pathogen-responsive MAPKs. Using a genetic approach, we identified additional ACS isoforms including ACS7, ACS8, and ACS11 that also contribute to pathogen-induced ethylene production. In addition to direct phosphorylation modification and stabilization of ACS2 and ACS6 proteins, MPK3 and MPK6 also regulate the expression of *ACS2* and *ACS6* genes through another MPK3/MPK6 substrate, WRKY33, a member of the plant-specific WRKY transcription factor family. Regulations of ACS isoforms at both transcriptional and post-translational levels contribute to the high-level ethylene production in plants challenged by invading pathogens. These findings shed light on our understanding of the regulation of the kinetics and magnitude of ethylene induction under different stress conditions.

## Introduction

The gaseous phytohormone ethylene profoundly impacts plant growth, development, and response to environmental stimuli [1]–[7]. Studies from a number of labs have defined a signaling pathway—from ethylene receptors to downstream signaling components to transcription factors—that alters gene expression and leads to ethylene-induced phenotypes (reviewed in [1], [2], [5], [8], [9]). Ethylene-regulated responses are suppressed in the absence of ethylene, and such suppression is released upon plant sensing of ethylene. As a result, all ethylene-regulated processes begin with the induction of ethylene biosynthesis [10]. Plants under stress, including wounding, flooding, drought, osmotic shock, ozone, and pathogen/insect invasion, produce elevated levels of ethylene [1], [6], [7], [11]. For this reason, ethylene is also known as a plant stress hormone. The biosynthetic pathway of ethylene has been fully elucidated for over two decades. Two enzymatic steps are unique to ethylene biosynthesis: conversion of *S*-adenosyl-methionine (SAM), a common metabolic precursor, to 1-amino-cyclopropane-1-carboxylic acid (ACC) by ACC synthase (ACS) and oxidative cleavage of ACC to form ethylene by ACC oxidase (ACO) [1], [4], [12]. ACS activity is very low in tissues that do not produce a large amount of ethylene and is enhanced under conditions that promote ethylene formation [1], [4], [12]–[14]. In contrast, ACO is constitutively present in most vegetative tissues. As a result, ACS is believed to be the committing and generally rate-limiting enzyme in ethylene biosynthesis.

ACS is encoded by a small gene family in plants. In Arabidopsis, there are nine ACS members. Based on the presence/absence of phosphorylation sites in their C-termini, ACS isoforms are classified into three types [15]. Type I ACS isoforms, which include Arabidopsis ACS1, ACS2, and ACS6, have phosphorylation sites by both mitogen-activated protein kinases (MAPKs) and calcium-dependent protein kinases (CDPKs) [16], [17]. Type II ACS isoforms, which include Arabidopsis ACS4, ACS5, ACS8, and ACS9, only have putative CDPK phosphorylation sites. In contrast, Type III ACS isoforms have shorter C-terminal extension and lack both phosphorylation sites. ACS7 and ACS11 are the two Type III ACS isoforms in Arabidopsis. ACS1 has a short deletion with the highly conserved tripeptide Thr-Asn-Pro (TNP) missing. It is enzymatically inactive as a homodimer, but can form functional heterodimers with other Type I isoforms and may contribute to ethylene biosynthesis [18], [19]. ACS isoforms show cell- and tissue-specific expression and are developmentally regulated. In addition, expression of some members is highly responsive to extracellular stimuli [1], [20].

More recent studies have highlighted the importance of ACS protein stability regulation by protein phosphorylation and dephosphorylation. MAPK cascades are signaling modules downstream of sensors/receptors that transduce extracellular stimuli into intracellular responses in eukaryotes. A basic MAPK cascade is composed of three interconnected kinases. MAPKs function at the bottom of the three-kinase cascade and are activated by MAPK kinases (MAPKKs) through phosphorylation on the Thr and Tyr residues in their activation motif between the kinase subdomain VII and VIII. The activity of MAPKKs is, in turn, regulated by MAPKK kinases (MAPKKKs) via phosphorylation of two Ser/Thr residues in the activation loop of MAPKKs. MAPKKKs receive signals from upstream receptors/sensors, most of the time indirectly with additional components involved [21], [22]. The outputs of a MAPK cascade are dependent on the substrates of the MAPK(s) in the cascade. A subset of MAPKs in plants, represented by tobacco SIPK/Ntf4/WIPK and Arabidopsis MPK3/MPK6, is activated under various stress conditions that elevate ethylene production (reviewed in [21], [23]–[26]). A gain-of-function analysis in tobacco revealed that activation of SIPK/WIPK induces high levels of ethylene production [27]. More detailed analyses in Arabidopsis have demonstrated that ACS2 and ACS6, two Type I ACS isoforms, are substrates of MPK3 and MPK6 [16], [28]. Phosphorylation of ACS2/ACS6 by MPK3 and MPK6 stabilizes the ACS protein *in vivo*, resulting in increases in cellular ACS activity and in ethylene production. The degradation machinery targets the C-terminal, non-catalytic domain of ACS6 and possibly ACS2 because of their sequence similarity [29]. Phosphorylation of ACS6 introduces negative charges to its C-terminus, which reduces the turnover of ACS6 by the ubiquitin-proteasome degradation machinery.

In addition to protein phosphorylation, protein dephosphorylation also plays critical role in ACS stability regulation. Recently, it was demonstrated that protein phosphatase 2A dephosphorylates ACS2/ACS6 and destabilizes them, a critical process that counteracts with MAPK phosphorylation [30]. Members of the Type II group, including ACS5 and ACS9, are also regulated at protein stability levels, possibly by protein phosphorylation as well [15], [31]–[33]. However, the kinase(s) involved remain unidentified. Because of the complex regulation of ACS protein/activity at multiple levels, many details about the up-regulation of ethylene biosynthesis remain unclear, including the specific ACS isoforms involved in the ethylene induction in response to a specific stimulus, the regulatory pathways that control the expression of *ACS* genes, and the components involved in the regulation of ACS protein stability. It has been known for decades that a subset of *ACS* genes, including Arabidopsis *ACS6*, is transcriptionally activated in plants under stress or pathogen attack. However, the importance of this transcriptional activation and the underlying regulatory mechanism are not known. Furthermore, ethylene induction by different stimuli exhibits different kinetics and magnitude. The underlying molecular mechanism of such differential induction is also unclear.

We are interested in the regulation of ethylene biosynthesis in plants infected by pathogens. ACS2 and ACS6, two Type I ACS isoforms, are involved in *Botrytis*-induced ethylene production [28]. The residual levels of ethylene induction in the *acs2/acs6* double mutant suggest involvement of additional ACS isoforms. In this study, we investigated (1) the potential involvement of all ACS isoforms in ethylene induction triggered by *B. cinerea* infection, (2) the importance of transcriptional activation of *ACS* gene expression, (3) the signaling pathways involved in the *ACS* gene activation, and (4) the molecular mechanism underlying the differential kinetics and magnitude of ethylene induction by different stimuli. We found that members in all three ACS groups are involved in pathogen-induced ethylene production, with ACS2, ACS6, and ACS7 contributing the most to *B. cinerea*-induced ethylene production. Based on analyses of an *ACS6* knockdown mutant and of conditional gain-of-function *ACS6* transgenic lines, we also can conclude that the transcriptional activation of the *ACS6* gene plays a critical role in sustaining high levels of ethylene induction. Interestingly, MPK3 and MPK6 not only function in the phosphorylation-induced stabilization of ACS2/ACS6 proteins, but also signal the *ACS2* and *ACS6* gene activation after *B. cinerea* infection. WRKY33, a MPK3/MPK6 substrate that regulates camalexin biosynthesis [34], is also responsible for turning on *ACS2/ACS6* expression downstream of MPK3/MPK6 cascade. WRKY33 binds to the W-boxes in the *ACS2/ACS6* promoters *in vivo* and is directly involved in MPK3/MPK6-induced *ACS2/ACS6* gene expression. The duration and magnitude of MPK3/MPK6 activation vary with different stimuli and correlate well with the duration and magnitude of ethylene induction. Regulation of ACS activity at multiple levels by the MPK3/MPK6 cascade is an important mechanism by which the levels/kinetics of ethylene production are regulated during plant stress/defense response.

## Results

### Activation of *ACS* gene expression in *B. cinerea*–infected Arabidopsis

**Figure 1:**
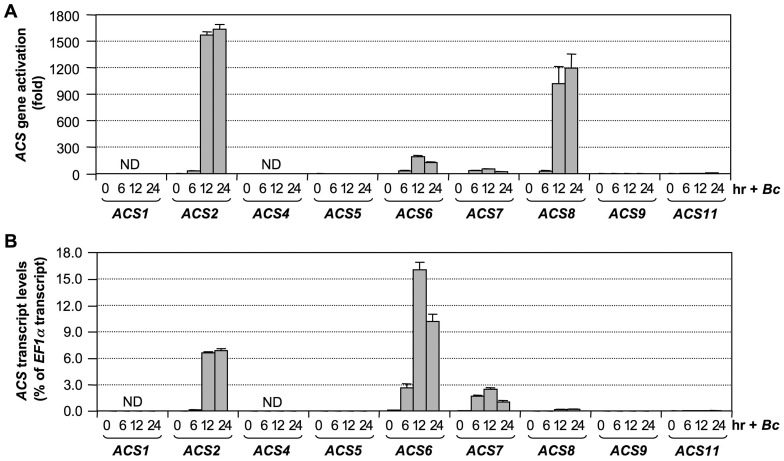
Activation of *ACS* gene expression in Arabidopsis after *B. cinerea* infection. (A) Twelve-day-old Arabidopsis seedlings grown in GC vials were inoculated with *B. cinerea* spores. Samples were collected at indicated times for total RNAs isolation. Expressions of all nine *ACS* genes were quantified by real-time PCR. Induction of *ACS* gene expression (fold of induction relative to the level before inoculation) was calculated by the double ΔCt method. (B) *ACS* transcript levels are expressed as percentage of *EF1α* transcript, which allows comparison of expression levels between different *ACS* genes. In both calculations, the expression of *EF1α* was used as a reference. Error bars indicate standard deviations (n = 3). ND, not detectable.

Our previous research demonstrated involvement of ACS2 and ACS6 in ethylene induction in *B. cinerea*-infected Arabidopsis [28]. This research also implicated the involvement of additional *ACS* genes since there was still an approximately 25% residual level of ethylene induction in the *acs2/acs6* double mutant. To identify the ACS isoforms involved, we profiled the expression of all nine *ACS* genes in Arabidopsis infected with *B. cinerea*. As shown in Figure 1A, transcripts of *ACS2*, *ACS6*, *ACS7*, and *ACS8* accumulated approximately 1600, 200, 50, and 1200 fold, respectively, over their basal levels. *ACS11* transcript also accumulated about 6 fold. *ACS5* and *ACS9* transcripts could be reliably detected, but no increases were observed. In contrast, *ACS1* and *ACS4* transcripts were not detectable. To better assess the potential contribution of each *ACS* gene to ethylene production, we also calculated their expression levels relative to that of *EF1α* (Figure 1B). This calculation allowed us to compare the relative levels of expression between different *ACS* genes. From this dataset, we found that the expression levels of *ACS2*, *ACS6*, and *ACS7* were among the highest. *ACS8* and *ACS11* had lower levels of expression after induction, while *ACS5* and *ACS9* expression remained very low. The expression of *ACS8* increased more than 1200 fold relative to its basal level (Figure 1A). However, because of its low basal level expression, the induced level of *ACS8* transcript was still much lower than those of *ACS2*, *ACS6*, and *ACS7* (Figure 1B). If regulation at other levels is the same, *ACS8* is likely a minor contributor despite the high-fold induction. In contrast, levels of *ACS7* transcript were considerably elevated (Figure 1B), despite a relatively low fold induction (Figure 1A), a result of a relatively high basal level. Based on these results, we speculated that *ACS7* might be a major contributor to ethylene induction after plant sensing of pathogen invasion besides *ACS2* and *ACS6*.

### *B. cinerea*–induced ethylene production involves ACS isoforms in all three groups

To establish the involvement of *ACS7*, we identified two null mutant alleles of *ACS7*, *acs7-1* (FLAG_431D05, in Ws-0 background) and *acs7-2* (CSHL_ET5768, in Ler-0 background). In both mutant alleles, *B. cinerea*-induced ethylene production is slightly reduced (Figure S1), similar to that in the *acs2* or *acs6* single mutant [28]. This result suggests that *ACS7* also contributes to *B. cinerea*-induced ethylene production. In our previous publications [16], [28], we did not assign allele numbers to the *acs2* and *acs6* mutants. To be consistent with the nomenclature used in Dr. Theologis's lab [35], the *acs6* allele (Salk_090423) was given an allele number of *acs6-2*. This allele turned out to be a knockdown mutant (more discussion later). In contrast, the *acs6-1* mutant allele (SALK_025672) in the study by Tsuchisaka et al. (2009) is a null mutant with a T-DNA insertion in the open reading frame (ORF) [35]. We failed to identify any plant with a T-DNA insertion when we initially ordered this line from the Arabidopsis Biological Resource Center (ABRC) in 2003. The *acs2* and *acs7-1* mutant alleles we used [16], [28](Figure S1) are the same as the *acs2-1* and *acs7-1* alleles, respectively, in Tsuchisaka et al. (2009) report [35].

**Figure 2:**
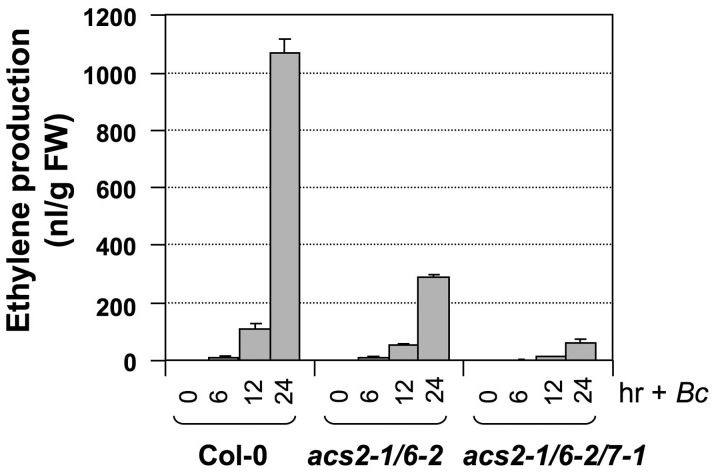
*ACS7* also contributes to *B. cinerea*-induced ethylene production in Arabidopsis. Twelve-day-old wild type (Col-0), *acs2-1/acs6-2* double mutant, and *acs2-1/acs6-2/acs7-1* triple mutant Arabidopsis seedlings grown in GC vials were inoculated with *B. cinerea*. Ethylene levels in the headspace were determined at indicated times. Error bars indicate standard deviations (n = 3).

**Figure 3:**
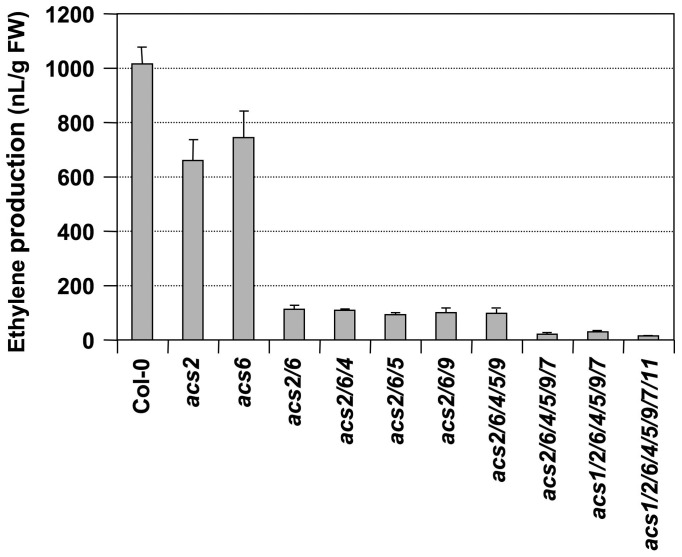
Ethylene induction in high-order *acs* mutants after *B. cinerea* infection. Twelve-day-old wild type (Col-0) and *acs* mutants generated in Dr. Theologis' lab were inoculated with *B. cinerea*. Ethylene levels in the headspace were determined at 24 hrs after spore inoculation. Error bars indicate standard deviations (n = 3). The allele numbers are omitted for easy labeling. They are *acs1-1*, *acs2-1*, *acs4-1*, *acs5-2*, *acs6-1*, *acs7-1*, *acs9-1*, and *acs11-1*.

We then crossed *acs7-1* into the *acs2-1/acs6-2* double mutant background and identified an *acs2-1/acs6-2/acs7-1* triple mutant in the F3 generation. As shown in Figure 2, only about 10% residual ethylene production was observed in the *acs2-1/acs6-2/acs7-1* triple mutant, confirming the importance of *ACS7* in *B. cinerea*-induced ethylene production. Residual ethylene induction in the *acs2-1/acs6-2/acs7-1* triple mutant again points to involvement of additional *ACS* members. To identify them, we utilized the high-order *acs* mutants generated in Dr. Theologis' lab [35]. We found that *acs2-1/acs6-1* seedlings produced a lower level of ethylene than our *acs2-1/acs6-2* double mutant after challenged with *B. cinerea* (Figure 3 and Figure S2), which is consistent with the knockdown nature of our *acs6-2* allele. Additional mutation of *ACS4*, *ACS5*, and *ACS9* genes, either one at a time or all three at once, in the *acs2-1/acs6-1* background did not further reduce the ethylene induction. This finding is consistent with our previous conclusion that *ACS5* and *ACS9* are not involved in the ethylene induction triggered by *B. cinerea* infection [28]. In contrast, mutation of the *ACS7* gene in the *acs2-1/acs6-1/acs4-1/acs5-2/acs9-1* background resulted in further reduction in the ethylene induction. In this sextuple mutant (*acs2-1/acs6-1/acs4-1/acs5-2/acs9-1/acs7-1*) background, mutation of *ACS11*, but not *ACS1*, slightly reduced the ethylene production. The very low level of ethylene induction in the *acs1-1/acs2-1/acs6-1/acs4-1/acs5-2/acs9-1/acs7-1/acs11-1* plants also implicates the involvement of *ACS8*. In the absence of *B. cinerea* infection, seedlings of all genotypes produced less than 15 nL ethylene per gram of seedlings within 24 hours, a very low level in comparison to the *B. cinerea*-induced ethylene production (Figure S3). From this dataset, we can also conclude that *ACS7* contributed the most to the basal level ethylene production. Seedlings without a functional *ACS7* gene including the *acs1-1/acs2-1/acs6-1/acs4-1/acs5-2/acs9-1/acs7-1/acs11-1* failed to produce a detectable level of ethylene. As a result, the low-level ethylene production in this octuple *acs* mutant in response to *B. cincerea* infection is indeed a contribution of *ACS8* gene.

In summary, we can conclude that *ACS2* and *ACS6* are major contributors of ethylene induction and that *ACS7*, *ACS8*, and *ACS11* contribute less, with a total of ∼15% of the ethylene induction in *B. cinerea*-infected Arabidopsis. Among the minor contributors, the role of *ACS7* and *ACS8* in *B. cinerea*-induced ethylene production is clear. In contrast, the contribution of *ACS11* is somewhat uncertain because of the conclusion of its involvement is based on a small quantitative difference. One potential mechanism underlying each ACS isoform's contribution to ethylene induction is through the up-regulation of their gene expression (Figure 1). There is a good correlation between the transcriptional activation of *ACS* gene expression (Figure 1) and involvement in *B. cinerea*-induced ethylene production (Figure 3).

### Induction of *ACS2* and *ACS6* gene expression is dependent on MPK3/MPK6 pathway

Previously, we demonstrated the importance of phosphorylation regulation of ACS2 and ACS6 by MPK3 and MPK6 in Arabidopsis in response to pathogens/pathogen-associated molecular patterns (PAMPs) [16], [28], [29]. It is well known that the *ACS6* gene is highly induced by stress, including wounding and pathogen infection [1], [4], [6], [11], [36], [37]. However, the importance of *ACS* gene activation in pathogen-induced ethylene production remains unclear. After our discovery that phosphorylation of ACS2 and ACS6 proteins by MPK3/MPK6 is required for ACS2/ACS6 protein stabilization and accumulation, we started to explore the potential contribution of *ACS* gene activation. Theoretically, an increase in *ACS* transcript levels is likely to increase the rate of *de novo* ACS protein synthesis, which, in turn, will increase the net ACS protein/activity after MAPK phosphorylation and protein stabilization.

**Figure 4:**
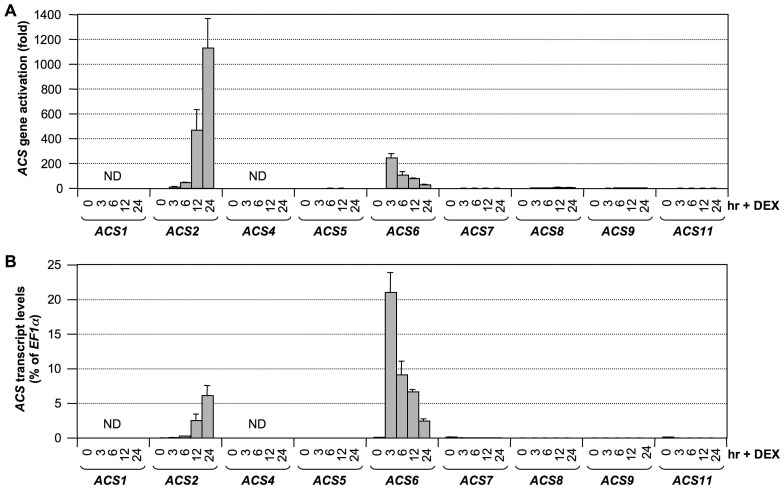
Activation of *ACS* gene expression after MPK3/MPK6 activation in the gain-of-function *DD* Arabidopsis seedlings. (A) Twelve-day-old conditional gain-of-function *DD* Arabidopsis seedlings grown in GC vials were treated with 1-µM DEX. Samples were collected at indicated times for total RNA preparation. After reverse transcription, expressions of all nine *ACS* genes were quantified by real-time PCR. Induction of *ACS* gene expression (fold of induction relative to the level before inoculation) was calculated by the double ΔCt method. (B) *ACS* transcript levels expressed as percentage of *EF1α* transcript, which allows comparison of expression levels among different *ACS* genes. In both calculations, the expression of *EF1α* was used as a reference. Error bars indicate standard deviations (n = 3). ND, not detectable.

To determine whether ethylene induction in the conditional gain-of-function *GVG-NtMEK2^DD^* (*DD*, for short) plants [16], [28] is associated with *ACS* gene activation, we profiled the expression of *ACS* genes in *DD* plants after dexamethasone (DEX) treatment. As shown in Figure 4, the expressions of *ACS2* and *ACS6* were highly induced. Different from *B. cinerea*-infected seedlings, no induction in *ACS7*, *ACS8*, and *ACS11* expression levels were observed. In addition, we noticed the kinetics of *ACS6* induction in the *DD* plants were different from that in *B. cinerea*-infected plants (Figure 4 versus Figure 1). This difference is likely a result of the synchronous response in *DD* plants. In contrast, the infection process of *B. cinerea* is progressive. As more cells sensed the growing hyphae, higher levels of *ACS6* transcript were induced at later time points.

**Figure 5:**
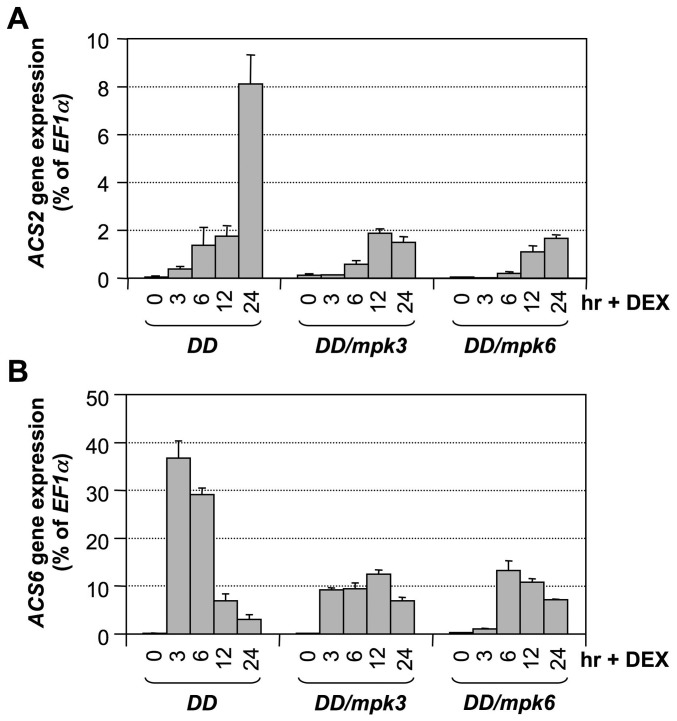
Activation of *ACS2* and *ACS6* gene expressions in gain-of-function *DD* is dependent on downstream *MPK3* and *MPK6*. Twelve-day-old *DD*, *DD/mpk3*, and *DD/mpk6* seedlings grown in GC vials were treated with 1-µM DEX. Samples were collected at indicated times. Total RNAs were extracted and treated with DNase to remove trace genomic DNA contamination. After reverse transcription, expressions of *ACS2* (A) and *ACS6* (B) genes were quantified by real-time PCR. *ACS* transcript levels were calculated as a percentage of the *EF1α* transcript. Error bars indicate standard deviations (n = 3).

To confirm that MPK3 and MPK6 are responsible for induction of *ACS2* and *ACS6* expression levels in the gain-of-function *DD* plants, we examined the expression of *ACS2* and *ACS6* in *DD/mpk3* and *DD/mpk6* plants. As shown in Figure 5, induction of *ACS2* and *ACS6* was compromised in both the *mpk3* and *mpk6* single mutant backgrounds. This finding demonstrates that induction of *ACS2* and *ACS6* in *DD* seedlings after DEX treatment is indeed a result of MPK3/MPK6 activation. Based on the fact that MPK3 and MPK6 are highly activated after *B. cinerea* infection [28], [38], we speculate that the MPK3/MPK6 cascade is involved in regulating the *B. cinerea*-induced *ACS2/ACS6* gene expression and that induction of *ACS7*, *ACS8*, and *ACS11* expression is regulated by pathway(s) other than MPK3/MPK6 cascade.

**Figure 6:**
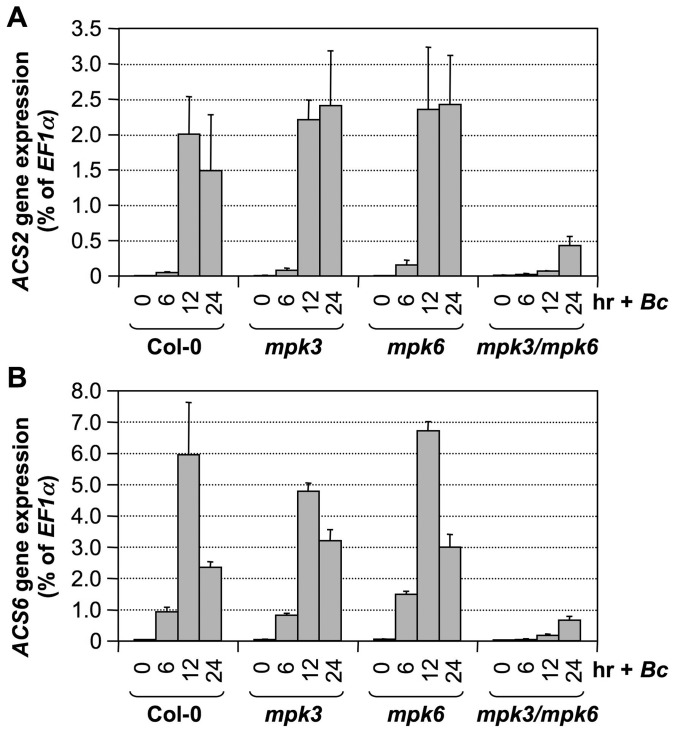
*B. cinerea* induced *ACS2* and *ACS6* gene activation is dependent on functional MPK3 and MPK6. Twelve-day-old wild type (Col-0), *mpk3*, *mpk6*, and rescued *mpk3/mpk6* double mutant seedlings grown in GC vials were inoculated with *B. cinerea* spores. Samples were collected at indicated times. Total RNAs were extracted and treated with DNase to remove trace genomic DNA contamination. After reverse transcription, expressions of *ACS2* (A) and *ACS6* (B) genes were quantified by real-time PCR. *ACS* transcript levels were calculated as a percentage of the *EF1α* transcript. Error bars indicate standard deviations (n = 3).

To provide loss-of-function evidence to support the role of the MPK3/MPK6 cascade in *B. cinerea*-induced *ACS2/ACS6* gene activation, we compared the induction of *ACS2* and *ACS6* gene expressions in wild type, *mpk3* single mutant, *mpk6* single mutant, and rescued *mpk3/mpk6* double mutant. The rescued *mpk3/mpk6* double mutant was obtained by transforming a DEX-inducible promoter-driven *MPK6* (*GVG-MPK6*) into *mpk3^−/−^/mpk6^+/−^* plants. When the T3 *mpk3^−/−^/mpk6^+/−^/GVG-MPK6^+/+^* plants began to flower, DEX was sprayed every other day to rescue the embryo lethality of the *mpk3^−/−^/mpk6^−/−^/GVG-MPK6^+/+^* zygotes. Progenies with *mpk3^−/−^/mpk6^−/−^/GVG-MPK6^+/+^* genotype were called rescued *mpk3/mpk6* double mutants [39], and were used for this experiment. As shown in Figure 6, *B. cinerea*-induced *ACS2* and *ACS6* expressions were little affected in either the *mpk3* or *mpk6* single mutant. In the rescued *mpk3/mpk6* double mutant, the induction of both genes was dramatically reduced, which supports the conclusion that MPK3 and MPK6 regulate expressions of *ACS2* and *ACS6* based on the gain-of-function analysis.

Different from *B. cinerea*-induced *ACS2/ACS6* gene activation, gain-of-function *DD*-induced *ACS2/ACS6* gene activation was compromised in either *mpk3* or *mpk6* single mutant background (Figure 5 and Figure 6). There are several potential explanations for this seemingly contradictory observation. First of all, MAPK-phosphorylation regulation of *ACS2/ACS6* gene activation will be affected by both the phosphorylation of the downstream transcription factor(s) such as WRKY33 (more discussion below), and their dephosphorylation by the unidentified phosphatase(s). It is possible that in the gain-of-function *DD* plants, both MPK3 and MPK6 are needed to overcome the action of the phosphatase(s) to maintain the phosphorylation of there transcription factor(s) and the subsequent up-regulation of *ACS2/ACS6* expression. In the absence of either MAPK, the signaling strength is below the threshold to counteract the phosphatases and the activation of *ACS2/ACS6* expression is severely compromised. It is possible that, in addition to the activation of MPK3/MPK6 cascade, pathogen infection may also inactivate the dephosphorylation process as a mechanism to promote higher levels of ethylene production. In this situation, the absence of only one MAPK may not be sufficient to block *ACS2/ACS6* activation. Alternatively, it is possible that the activation of pathways other than MAPK cascade can compensate the weakened MAPK pathway in the single *mpk3* or *mpk6* mutant, making it necessary to mutate both *MPK3* and *MPK6* to see the loss-of-function phenotype in response to *B. cinerea* infection. Similar phenomenon was also observed in MPK3/MPK6-mediated camalexin induction in response to *B. cinerea* infection [38].

### WRKY33 is involved in the MPK3/MPK6-regulated *ACS2/ACS6* gene activation

**Figure 7:**
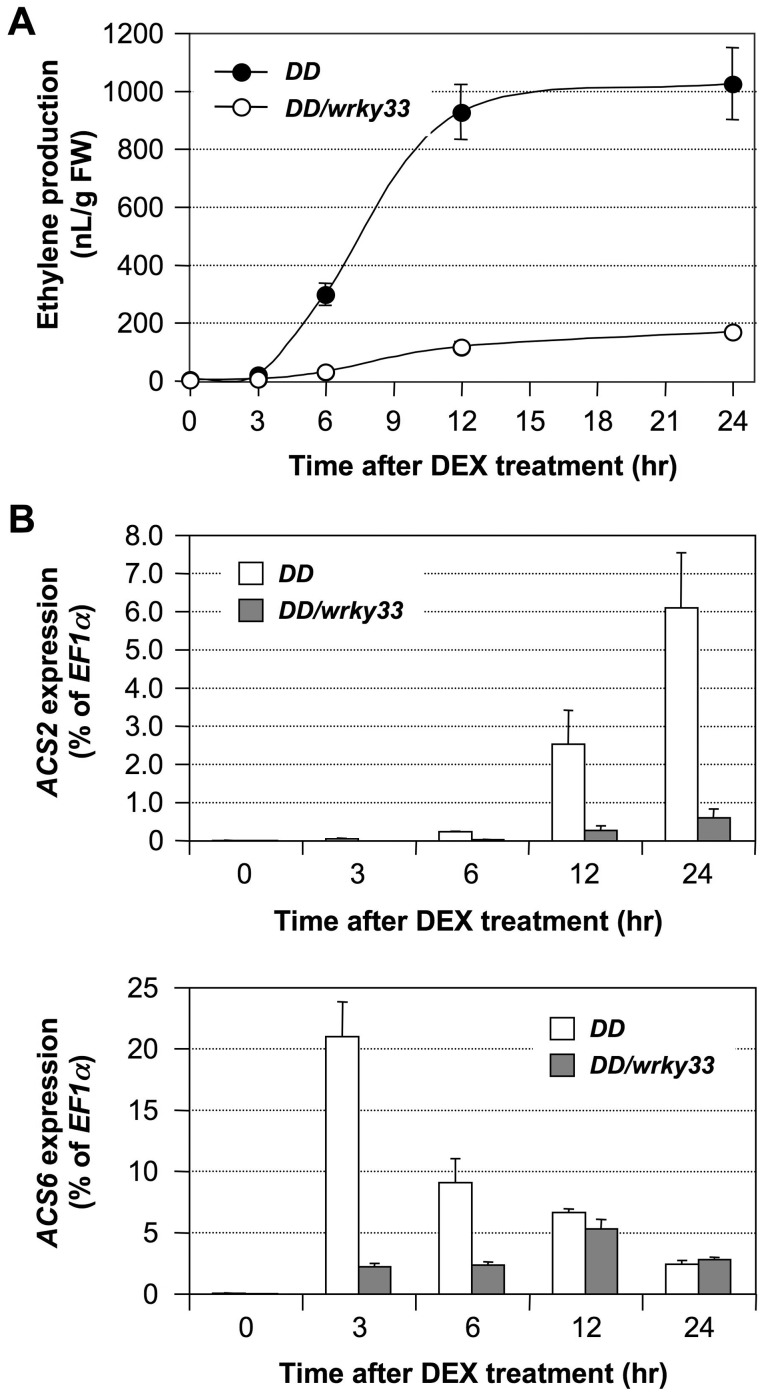
*WRKY33* functions downstream of the MPK3/MPK6 cascade in inducing the expression of *ACS2* and *ACS6* genes in the gain-of-function *DD* seedlings. (A) Mutation of *WRKY33* compromises ethylene induction in *DD* seedlings. Twelve-day-old *DD* and *DD/wrky33* seedlings grown in GC vials were treated with 1-µM DEX. Ethylene accumulation in GC vials was monitored at indicated times, and then seedlings were collected for gene expression analysis. Error bars indicate standard deviations (n = 3). (B) MPK3/MPK6-induced *ACS2* and *ACS6* gene expression in *DD* plants is dependent on *WRKY33*. Total RNA was extracted from seedlings collected in (A). Expressions of *ACS2* (upper panel) and *ACS6* (lower panel) genes were quantified by real-time PCR. *ACS* transcript levels were calculated as a percentage of the *EF1α* transcript. Error bars indicate standard deviations (n = 3).

WRKY33 is a substrate of MPK3/MPK6 in regulating the pathogen-induced phytoalexin biosynthesis [34]. WRKY33 functions as a transcriptional activator downstream of MPK3 and MPK6 in promoting the expression of camalexin biosynthetic genes. To determine whether WRKY33 also is involved in activation of *ACS2* and *ACS6* genes downstream of the MPK3/MPK6 cascade, we quantified the expression of these two genes in *DD* and *DD/wrky33* plants. As shown in Figure 7B, the induction of *ACS2* and *ACS6* mRNA by the gain-of-function *DD* transgene was compromised in *wrky33* mutant background. Associated with this, the induction of ethylene biosynthesis was mostly inhibited (Figure 7A). Previously, we showed that *DD* protein induction and MPK3/MPK6 activation in *DD/wrky33* plants are indistinguishable from those in *DD* plants [34], which strongly supports the conclusion that WRKY33 also functions downstream of MPK3/MPK6 in promoting the expression of *ACS2* and *ACS6* genes. The low residual levels of *ACS2* and *ACS6* gene activation is likely to be a result of other WRKY transcription factors that can partially substitute for the loss of *WRKY33*.

**Figure 8:**
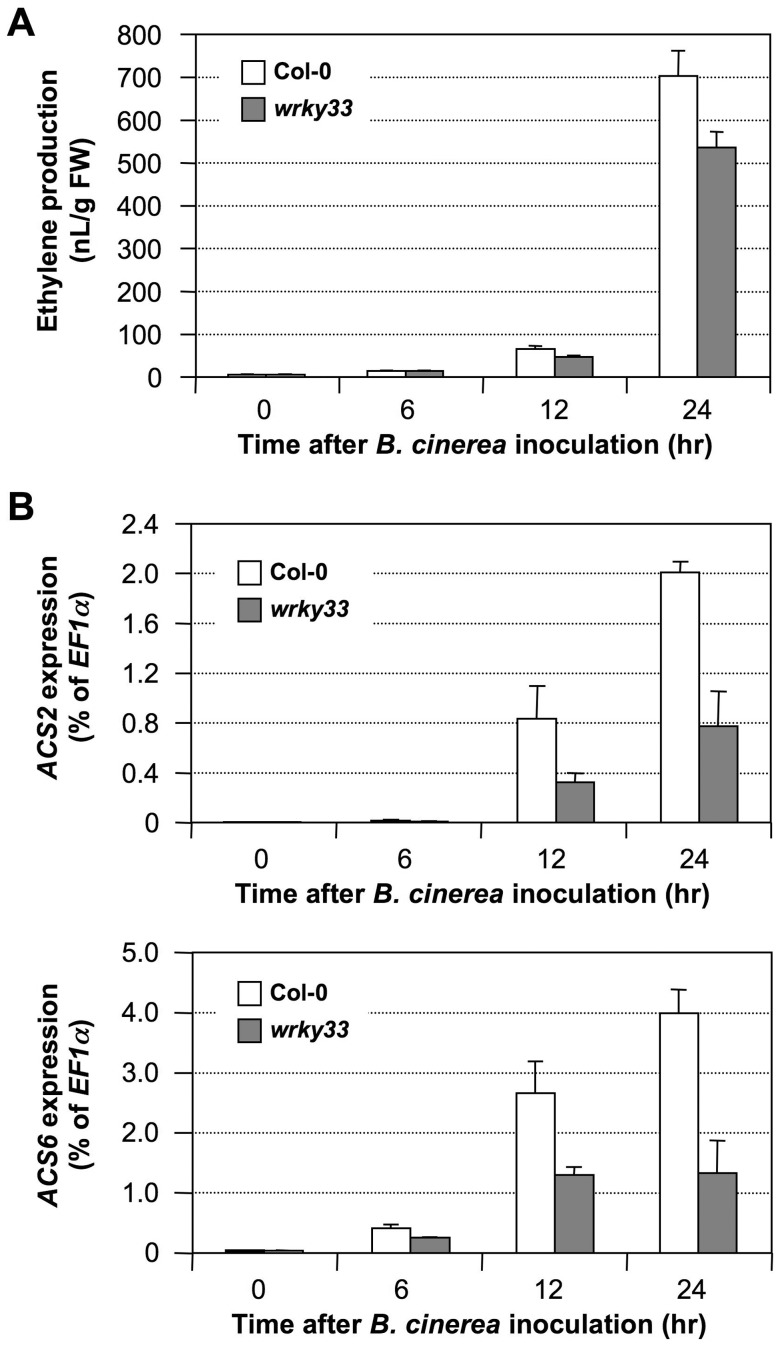
Induction of *ACS2* and *ACS6* gene expression after *B. cinerea* infection was partially inhibited in *wrky33* mutant. (A) Mutation of *WRKY33* partially blocks ethylene induction in Arabidopsis infected by *B. cinerea*. Twelve-day-old wild type (Col-0) and *wrky33* seedlings grown in GC vials were inoculated with *B. cinerea* spores. Ethylene accumulation in GC vials was monitored at indicated times, and seedlings were collected for gene expression analysis. Error bars indicate standard deviations (n = 3). (B) *B. cinerea*-induced *ACS2* and *ACS6* gene expression is compromised in the *wrky33* mutant. Total RNA was isolated from the seedlings collected in (A). Expressions of *ACS2* (upper panel) and *ACS6* (lower panel) genes were quantified by real-time PCR. *ACS* transcript levels were calculated as a percentage of the *EF1α* transcript. Error bars indicate standard deviations (n = 3).

In contrast to the gain-of-function *DD* plants, mutation of the *WRKY33* gene had a minor impact on ethylene induction triggered by *B. cinerea* infection. As shown in Figure 8A, we observed only about a 20% decrease in ethylene production in both alleles of the *wrky33* mutant. A comparison of *ACS2* and *ACS6* gene expressions in the wild type and in the *wrky33* mutant revealed that about one-third of the induction in *ACS2/ACS6* expression remained in the *wrky33* mutant (Figure 8B). This suggests that *ACS2* and *ACS6* still could be partially activated in the absence of *WRKY33*. Because of the low residual *ACS2/ACS6* gene activation in the *mpk3/mpk6* mutant (Figure 6), we speculate that the residual levels seen in the *wrky33* mutant are MPK3/MPK6-dependent but WRKY33-independent, again pointing to additional transcription factors, possibly WRKY33 homologs that partially replace WRKY33 in its absence.

No major reductions were observed in the induction of *ACS7*, *ACS8*, and *ACS11* expression in *wrky33* infected with *B. cinerea* (Figure S4), which is consistent with the conclusion that their activation is MPK3/MPK6 and WRKY33 independent. The normal activation of *ACS7*, *ACS8*, and *ACS11* expressions, together with the residual level of *ACS2/ACS6* gene activation and protein phosphorylation stabilization, may explain the observation that the induction of ethylene in the *wrky33* mutant was reduced by only about 20% after *B. cinerea* infection (Figure 8A). In contrast, the *wrky33* mutation almost completely blocked induction of ethylene biosynthesis in the gain-of-function *DD* plants (Figure 7A). *B. cinerea* can activate multiple signaling pathways in plants. It is possible that pathway(s) other than MPK3/MPK6 cascade are able to partially compensate the loss of *WRKY33*. It is known that pathogen infection induces a large number of *WRKY* genes [40], [41], some of which might be able to partially compensate the loss of *WRKY33* gene in activating the expression of *ACS2/ACS6*.

### WRKY33 binds to the W-boxes in the promoters of *ACS2* and *ACS6* genes *in vivo*

**Figure 9:**
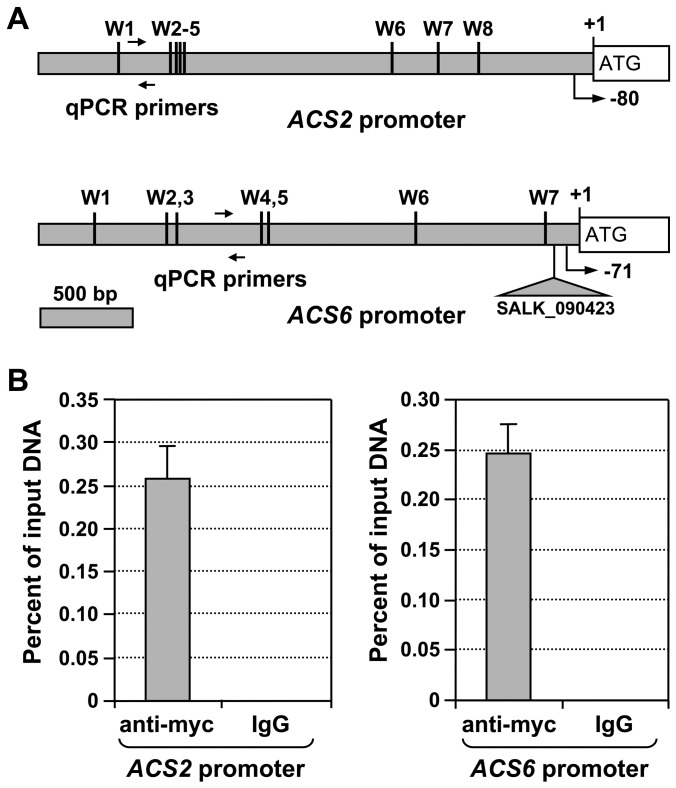
WRKY33 transcription factor binds to the promoter of *ACS2* and *ACS6* genes *in vivo*. (A) The promoters of *ACS2* and *ACS6* genes are rich in W-boxes, the cis-element binding sites of the WRKY transcription factor. A diagram indicates the number and relative position of the W-boxes in the promoters of the *ACS2* and *ACS6* genes. Line arrows indicate the position of primers used for qPCR after chromatin immunoprecipitation (ChIP). Positions of the predicted transcriptional starting sites are indicated by arrows with turning lines and negative numbers. The T-DNA insertion site in the SALK_090423 *acs6-2* allele, which locates in the promoter region of *ACS6* gene, is also indicated. (B) ChIP-qPCR analysis was performed using *DD/4myc-WRKY33^WT^* plants generated from the cross of *wrky33/4myc-WRKY33^WT^* with *DD* lines. Input chromatin was isolated from two-week-old seedlings 12 hr after DEX treatment. Epitope-tagged WRKY33-chromatin complex was immunoprecipitated with an anti-myc antibody. A control reaction was processed side-by-side using mouse IgG. ChIP- and input-DNA samples were quantified by real-time qPCR using primers specific to the promoters of *ACS2* (left panel) and *ACS6* (right panel) genes. ChIP results are presented as percentage of input DNA. Error bars indicate standard deviations (n = 3).

Genetic analysis revealed that *WRKY33* is essential for gain-of-function MPK3/MPK6-induced *ACS2/ACS6* gene expression (Figure 7B). Examination of the *ACS2* and *ACS6* promoters revealed the presence of eight and seven W-boxes, respectively (Figure 9A). To further substantiate the role of WRKY33 in the activation of *ACS2* and *ACS6* gene expression, we performed chromatin immunoprecipitation-quantitative PCR (ChIP-qPCR) analysis to determine whether *ACS2* and *ACS6* genes are direct targets of the WRKY33 transcription factor. For this experiment, we used *DD/wrky33* mutant plants complemented with a *35S* promoter-driven *WRKY33* transgene, which contains a four-copy myc epitope tag at the N-terminus (*DD/wrky33/35S:4myc-WRKY33*) [34]. The presence of the myc tag allowed us to immunoprecipitate the WRKY33-DNA complex by using a commercial anti-myc antibody. As shown in Figure 9B, immunoprecipitation with the anti-myc antibody greatly enriched *ACS2* and *ACS6* promoter regions containing the W-boxes. In contrast, the IgG control antibody failed to enrich either gene promoter. This result demonstrates that WRKY33 directly binds to the promoters of *ACS2* and *ACS6 in vivo*, suggesting that WRKY33 is the transcription factor downstream of the MPK3/MPK6 cascade involved in the activation of *ACS2* and *ACS6* expression.

### High levels of *ACS6* gene expression elevate *B. cinerea*–induced ethylene production

**Figure 10:**
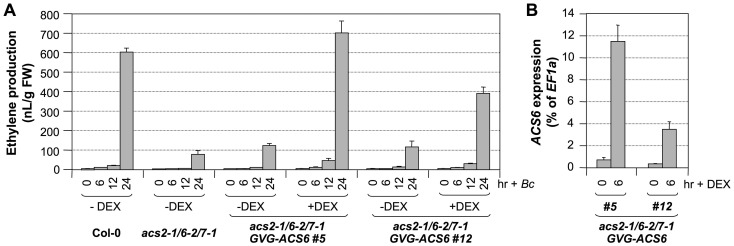
Conditional expression of the *ACS6* gene in the *acs2-1/acs6-2/acs7-1* mutant background restores ethylene induction triggered by *B. cinerea* infection. (A) Induction of *ACS6* gene expression in the *acs2-1/acs6-2/acs7-1/GVG-ACS6* seedlings restores *B. cinerea*-induced ethylene production. Twelve-day-old seedlings of wild type, *acs2-1/acs6-2/acs7-1*, and two independent lines of the *GVG-ACS6* transgene in the *acs2-1/acs6-2/acs7-1* background (#5 and #12) were inoculated with *B. cinerea* spores. Two groups of *acs2-1/acs6-2/acs7-1/GVG-ACS6* seedlings were included with one treated with 1-µM DEX and the other treated with an equal volume of ethanol, the solvent for the DEX stock solution, at the time of *B. cinerea* spore inoculation. Ethylene accumulation in GC vials was monitored at indicated times. Error bars indicate standard deviations (n = 3). (B) Induction of *ACS6* transgene expression in *acs2-1/acs6-2/acs7-1/GVG-ACS6* seedlings after DEX treatment. Seedlings were collected before (0 hr) and 6 hr after DEX treatment. Induction of the *ACS6* from the *GVG-ACS6* transgene was quantified by real-time PCR. *ACS6* transcript levels were calculated as a percentage of the *EF1α* transcript. Error bars indicate standard deviations (n = 3).

To provide further direct evidence in support of the role of *ACS6* gene activation in *B. cinerea*-induced ethylene production, we transformed a DEX-inducible promoter-driven *ACS6* (*GVG-ACS6*) construct into the *acs2-1/acs6-2/acs7-1* mutant background and then compared the ethylene induction in *acs2-1/acs6-2/acs7-1/GVG-ACS6* plants with and without DEX treatment. Two independent lines (#5 and #12) with different levels of *ACS6* transgene induction after DEX treatment were used for this experiment to establish a correlation between the levels of *ACS6* gene expression and the levels of ethylene induction. As shown in Figure 10A, without DEX treatment, both *GVG-ACS6* transgenic lines produced about the same levels of ethylene after *B. cinerea* treatment in comparison to the *acs2-1/acs6-2/acs7-1* triple mutant. In the presence of DEX, which induced *ACS6* expression (Figure 10B), the ethylene production was greatly enhanced. The higher level of *ACS6* induction in line #5 in the presence of DEX correlated with a higher level of ethylene induction than that in line #12. Furthermore, ethylene induction in Line #5 was higher than that in the wild type, indicating that transgene induction after DEX treatment not only complements the loss of *ACS6*, but also compensates the loss of *ACS2* and *ACS7* genes. In the absence of *B. cinerea* infection, DEX treatment only slightly elevated the ethylene production (Figure S5) to a level similar to the basal level ethylene production of Col-0 (∼10 nL/g FW in 24 hrs). This low-level ethylene production is likely a result of high-level *ACS6* gene induction after DEX treatment (and associated higher-level of *de novo* ACS6 protein synthesis) in combination with the basal level activity of MPK6, which can phosphorylate and stabilize ACS6 protein. MPK6 has very low basal activity even in the absence of stress/pathogen infection [16]. This is consistent with our previous conclusion that the overexpression of *ACS6* gene in the absence of MPK3/MPK6 activation is not sufficient to induce ethylene production due to the lack of phosphorylation stabilization [16]. As a result, we can conclude that the high level of ethylene production seen in *acs2-1/acs6-2/acs7-1/GVG-ACS6* lines after DEX and *B. cinerea* treatment is a combination of high level of gene expression (as a result of DEX treatment), and phosphorylation stabilization due to MPK3/MPK6 activation by *B. cinerea* infection.

**Figure 11:**
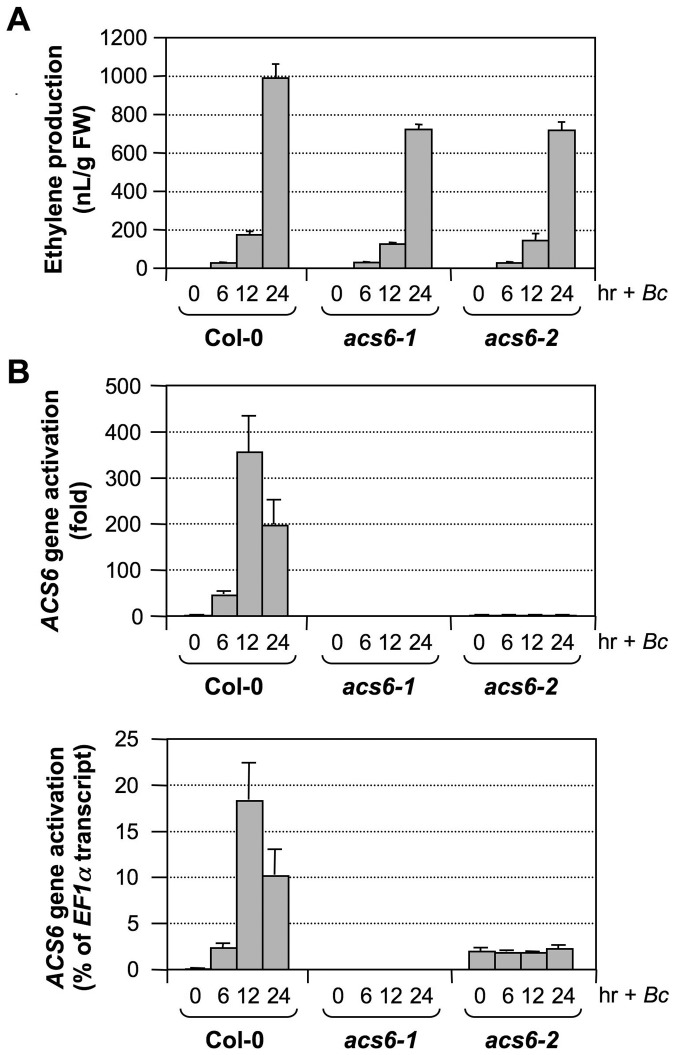
The *acs6-2* mutant allele is a knockdown mutant. (A) *B. cinerea*-induced ethylene production in wild type, *acs6-2* (SALK_090423), and *acs6-1* (SALK_025672) plants. Twelve-day-old seedlings grown in GC vials were inoculated with *B. cinerea* spores. Ethylene accumulation in GC vials was monitored at indicated times, and seedlings were collected for gene expression analysis. Error bars indicate standard deviations (n = 3). (B) Induction of *ACS6* expression in wild type (Col-0), *acs6-2*, and *acs6-1* seedlings after *B. cinerea* inoculation. Total RNA was isolated from the seedlings collected in (A). Expression of the *ACS6* gene was quantified by real-time PCR. *ACS6* transcript levels were expressed as fold of induction relative to the zero time point (upper panel) and as a percentage of the *EF1α* transcript (lower panel). Error bars indicate standard deviations (n = 3).

Our attempts to identify the T-DNA insertion line in the coding region of the *ACS6* gene (SALK_025672, *acs6-1*) failed to reveal a true mutant plant from the seeds received. As a result, we have been using the SALK_090423 line (*acs6-2*), which has a T-DNA insertion 170 bp upstream of the ATG start codon (Figure 9A)[16], [28]. In the past, we routinely used the double ΔCt method to quantify gene expression in real-time PCR analysis, which indicated the *acs6-2* mutant allele as a knockout mutant (Figure 11B, upper panel). However, a more careful analysis performed in this study revealed that it is actually a knockdown mutant with an elevated basal level expression. As shown in Figure 11B (lower panel), *acs6-2* seedlings showed a higher basal level of *ACS6* expression, but no increase in its transcripts was detected after *B. cinerea* infection. In contrast, no transcript was detected in the *acs6-1* mutant before and after *B. cinerea* infection. Side-by-side comparison demonstrated that ethylene production levels in *acs6-2* and *acs6-1* single mutants after *B. cinerea* inoculation were similar (Figure 11A). In contrast, *acs2-1/acs6-2* seedlings produce higher levels of ethylene than *acs2-1/acs6-1* (both have the same *acs2* mutant allele)(Figure S2). The observable difference between *acs6-2* and *acs6-1* alleles in the *acs2-1* mutant background could be due to the reduction of total ethylene production in the absence of *ACS2* gene, which makes it possible to observe a small difference. These results suggest that *acs6-2* mutant allele is not a null mutant as *acs6-1* allele, and that the high level induction of *ACS6* is important to pathogen-induced ethylene production. Together with the gain-of-function evidence shown in Figure 10, we can conclude that *ACS6* gene activation plays an essential role in promoting ethylene production in plants challenged by pathogens.

## Discussion

Plants challenged by pathogens, especially necrotrophs such as *B. cinerea*, produce very high levels of ethylene, a critical event in plant disease resistance [6], [7], [35]. In contrast, other stress stimuli, including wounding, only trigger a transient and low-level ethylene biosynthesis. Previously, we reported that MPK3/MPK6 phosphorylation-induced stabilization of ACS2 and ACS6 proteins is an important mechanism in promoting ethylene induction in Arabidopsis [16], [28], [29]. It is also clear from data based on *acs2-1/acs6-2* double mutant that ACS2 and ACS6 are not the only contributors in pathogen-induced ethylene production [28]. In this report, we demonstrate the involvement of three additional members of the *ACS* gene family. Mutation of *ACS2* and *ACS6*, two Type I ACS members, abolishes ∼85% of the ethylene production induced by *B. cinerea* (Figure 2 and Figure 3) [28]. We failed to detect the transcript from *ACS1*, the third member of the Arabidopsis Type I ACS isoforms, in the seedlings, and its mutation does not reduce pathogen-triggered ethylene production (Figure 3) [28]. As a result, we believe that *ACS1* contributes little, if any, to pathogen-triggered ethylene production. ACS7, a member of the Type III ACS, plays an intermediate role. Its mutation in the *acs2-1/acs6-2* background reduces ethylene induction to less than 5% of that observed in the wild type (Figure 2 and Figure 3). This isoform is also the major contributor of the basal level ethylene production in the absence of pathogen infection (Figure S3). The transcripts of *ACS2*, *ACS6*, and *ACS7* are among the most abundant in *B. cinerea*-infected Arabidopsis (Figure 1B). The residual levels of ethylene induction in *acs1-1/acs2-1/acs6-1/acs4-1/acs5-2/acs9-1/acs7-1* mutant are likely from *ACS8*, a Type II ACS isoform, and *ACS11*, a Type III ACS isoform.

### Dual-level regulation of ACS2 and ACS6 by the MPK3/MPK6 cascade in plant stress/defense response

**Figure 12:**
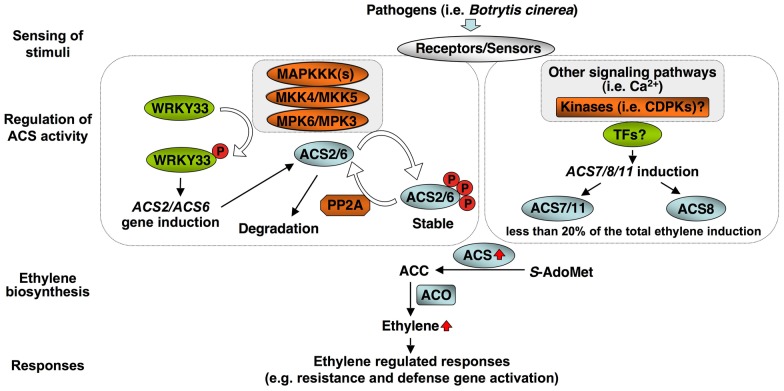
A model depicting the dual-level regulation of ACS activity by MPK3/MPK6-dependent and independent pathways during pathogen-induced ethylene production. Members of all three types of ACS isoforms are involved in pathogen-induced ethylene production. In *B. cinerea*-infected plants, Type I (ACS2/ACS6) isoforms contribute the most (∼85%). ACS2 and ACS6 are regulated by the MPK3/MPK6 cascade at both transcriptional and protein stability levels. The transcriptional up-regulation is mediated by WRKY33, a MPK3/MPK6 substrate. Type II (ACS8 and ACS11) and Type III (ACS7) isoforms are activated at the transcriptional level although the regulatory pathway(s) involved is not clear at present. Increase in total cellular ACS activity drives the elevated ethylene production, which triggers downstream responses.

In addition to post-translational regulation, we found that transcriptional activation of the *ACS* genes is also critical to the high-level of ethylene induction, as depicted in our working model (Figure 12). Stress- and pathogen-activation of *ACS* genes, such as Arabidopsis *ACS6*, is well established [1], [36], [42]. In this report, we delineated a signaling pathway involved in the transcriptional activation of *ACS2/ACS6* in Arabidopsis after pathogen infection. It is interesting to find that MPK3 and MPK6 not only function in the phosphorylation-induced stabilization of ACS2/ACS6 proteins, but also regulate the expression of *ACS2* and *ACS6* genes. The MPK3/MPK6 cascade-induced *ACS2/ACS6* gene activation is mediated by WRKY33, another MPK3/MPK6 substrate [34]. WRKY33 binds to the W-boxes in the promoters of the *ACS2* and *ACS6* genes directly *in vivo* (Figure 9) and is involved in the MPK3/MPK6-induced *ACS2/ACS6* gene expression (Figure 7). Mutation of *WRKY33* resulted in a smaller reduction (∼60%) in *ACS2/ACS6* gene activation in response to *B. cinerea* infection (Figure 8), possibly due to the presence of other WRKY(s) that can partially compensate the loss of *WRKY33*. Conditional overexpression of *ACS6* in the *acs2-1/acs6-2/acs7-1* mutant background greatly enhances the ethylene induction (Figure 10). Furthermore, reduction in ethylene induction in the *acs6-2* allele, a knockdown mutant (Figure 11), provides loss-of-function evidence that demonstrates the importance of *ACS6* gene activation during pathogen invasion. Transcriptional activation of *ACS2* likely has a similar role.

Induction of *ACS6* expression is associated with stress-induced ethylene production [1], [20], [36], [42]. However, direct evidence supporting the role of *ACS* gene activation has been lacking. In Arabidopsis, overexpression of wild type *ACS6* genes is not sufficient to elevate ethylene production because of the requirement of protein phosphorylation and stabilization [16]. In addition, overexpression of the *ACS6* gene in the wild type background fails to enhance ethylene production upon *B. cinerea* inoculation (Li, G., Liu, Y., and Zhang, S., unpublished data), a result of the high-level gene activation of the endogenous *ACS* genes (Figure 1). In this study, we expressed the *ACS6* gene in an *acs2-1/acs6-2/acs7-1* mutant background. The use of a DEX-inducible promoter and two independent lines with different levels of *ACS6* gene induction after DEX treatment allowed us to demonstrate the importance of *ACS6* gene activation (Figure 10).

Our *acs2-1/acs6-2* double mutant produces about 25% of the wild type level of ethylene after *B. cinerea* infection (Figure 2) [28]. In contrast, the *acs2-1/acs6-1* line only produces ∼15% of the wild type ethylene (Figure 3). The difference between these two double mutants is likely a result of different *acs6* mutant alleles since both have the same *acs2-1* mutant allele. The difference between *acs2-1/acs6-2* and *acs2-1/acs6-1* also suggests that the *acs6-2* allele is not a complete null mutant, which is supported by the presence of *ACS6* transcript in *acs6-2* allele (Figure 11B). Since the T-DNA insertion in this allele is in front of the transcriptional starting site (Figure 9A), functional transcript is likely to be produced in this mutant allele. Nonetheless, the reduction of ethylene induction in the *acs6-2* single mutant or in the *acs2-1/acs6-2* double mutant [28](Figure 2) demonstrates the importance of high-level induction of *ACS6* expression in pathogen-induced ethylene production. Interestingly, the reduction in ethylene induction in the *acs2/acs6* double mutant is always more than the sum of the reduction in each single mutant (Figure 3) [28]. It is possible that the heterodimers of ACS2 and ACS6 are less active than each of homodimer. In the absence of one isoform, only the homodimer can be formed, which partially compensates for the loss of the other isoform.

### Importance of dual-level regulation ACS isoforms in determining the levels and kinetics of ethylene induction

Although very inefficient energy-wise, regulation of the ACS protein at the protein stability level by phosphorylation/dephosphorylation allows rapid induction of ethylene biosynthesis, which can occur within minutes after plant sensing of external stimuli [43]–[45]. Such rapid response could be important to plant response to the stress/pathogen stimuli. However, even after phosphorylation stabilization, the ACS6 protein may not be very stable. The half-life of the phospho-mimicking ACS6^DDD^ is only ∼3 hours [29]. In the meantime, protein phosphatase 2A will counteract with the MAPKs by dephosphorylating the phospho-ACS protein [30]. Under this circumstance, transcriptional activation can provide another mechanism to further enhance the ethylene induction in response to pathogen infection. It is well known that stress-induced ethylene production follows different kinetics depending on the stimuli. However, the molecular mechanism underlying this difference is unclear. A good correlation exists between the kinetics of MPK3/MPK6 activation and ethylene induction. For instance, wounding induces a transient ethylene production, which is associated with a transient activation of MAPKs [46], [47]. In contrast, infection of plants by pathogens, especially necrotrophic fungal pathogens, triggers a long-lasting and high-level induction of ethylene biosynthesis, which correlates with a long-lasting and high-magnitude activation of MPK3/MPK6 [28].

As depicted in Figure 12, MPK3 and MPK6 regulate ethylene induction via two different mechanisms: by direct phosphorylation and stabilization of ACS2 and ACS6 proteins [16], [28], [29] and by activation of *ACS2* and *ACS6* gene expression (this study). Transient activation of MPK3/MPK6 by wounding is also associated with the activation of *ACS2/ACS6* gene expression [36]. However, due to the transient nature of MAPK activation, which returns to basal level within ∼0.5 hr to 1 hr [47], the *de novo* synthesized ACS protein may not have the chance to be phosphorylated and will be degraded quickly in the absence of MAPK phosphorylation. In *B. cinerea*-infected plants, the induction of *ACS2* and *ACS6* gene expression will result in high rates of *de novo* protein synthesis. On top of this, the high-level and long-lasting activation of MPK3/MPK6 [28], [38] can maintain *de novo* synthesized ACS2 and ACS6 proteins in a phosphorylated state and thereby stabilize the protein against proteasome-mediated degradation [16], [29]. This dual-level regulatory mechanism can maintain a greatly enhanced level of cellular ACS activity and ethylene production in pathogen-infected plants. Recently, it was shown that a PP2A protein phosphatase can counteract with MPK3/MPK6 by dephosphorylating ACS2/ACS6 and can destabilize the ACS protein [30]. In this situation, it is even more important to have the high-level, long-lasting activation of MPK3/MPK6 in order to maintain the ACS2/ACS6 protein in a phosphorylated state to ensure the high rate ethylene biosynthesis observed in plants challenged by pathogens.

### WRKY33 is a key transcriptional regulator downstream of MPK3/MPK6 in regulating gene expression in multiple pathways

Activation of MPK3/MPK6 and their orthologs in other plant species induces the expression of large number of stress/defense related genes [48], [49], suggesting the involvement of downstream transcription factors. ERF104 is a substrate of MPK6. Phosphorylation of ERF104 by MPK6 results in release of ERF104 from the complex, which allows ERF104 to activate the expression of genes further downstream [50]. Recently, we identified the transcription factor WRKY33 as the substrate of MPK3 and MPK6 [34]. WRKY33 is involved in the induction of camalexin biosynthesis by promoting the expression of camalexin biosynthetic genes [34], [51]. In this report, we demonstrate that WRKY33 is involved also in activation of *ACS2* and *ACS6* gene expression and ethylene induction. In the *wrky33* mutant background, gain-of-function *DD*-induced *ACS2* and *ACS6* gene activation is essentially abolished. Furthermore, a ChIP-qPCR analysis demonstrated that WRKY33 directly binds to the promoters of *ACS2* and *ACS6* genes (Figure 9). These results reveal that WRKY33 regulates gene expression in multiple stress/defense responses and may function as a master transcriptional regulator downstream of the MPK3/MPK6 cascade.

The expression of both *ACS2* and *ACS6* genes are regulated by the MPK3/MPK6 cascade and its downstream WRKY33. However, the induction kinetics of *ACS2* and *ACS6* genes are different in both gain-of-function *DD* transgenic plants (Figure 4) and wild type plants after pathogen treatment (Figure 1). One possibility is that one or more transcription factors, other than WRKY33, are involved. The differential involvement of these unknown transcription factors could result in the different kinetics observed in the induction of *ACS2* and *ACS6* genes. These transcription factors may or may not be regulated by the MPK3/MPK6 cascade. The activation of MPK3 and MPK6 proteins in the gain-of-function *DD* plants is sufficient to induce the expression of *ACS2* and *ACS6* genes to levels similar to those observed in *B. cinerea*-infected plants (Figure 1 and Figure 4), suggesting that the transcriptional machinery controlling expression of *ACS2* and *ACS6* genes is fully turned on in *DD* plants. On the other hand, mutation of *WRKY33* essentially blocks *DD*-induced *ACS2/ACS6* gene activation (Figure 7B) but only partially blocks the induction of *ACS2* and *ACS6* genes in *B. cinerea*-infected plants (Figure 8B), suggesting the activation of additional components by *B. cinerea* that cannot be activated by MPK3/MPK6 cascade alone, possibly homologs of WRKY33 that can partially replace the function of *WRKY33* in its absence.

### Contribution of MPK3/MPK6-independent pathway(s) to stress-/pathogen-induced ethylene biosynthesis

The aforementioned discussions are focused on the role of the MPK3/MPK6 cascade in regulating ACS2 and ACS6, two major contributors of pathogen-induced ethylene production, as depicted in our working model (Figure 12). A similar level of reduction in ethylene induction in the *mpk3/mpk6* and *acs2/acs6* double mutants (∼85%) is consistent with our conclusion that the MPK3/MPK6 signaling cascade only controls ACS2 and ACS6. Genetic evidence also supports the involvement of three additional ACS isoforms, ACS7, ACS8, and ACS11, in *B. cinerea*-induced ethylene production (Figure 2 and Figure 3). ACS7 and ACS11 are the two members in the Type III ACS group in Arabidopsis. ACS8 belongs to the Type II ACS group. Based on mutant analyses, these three *ACS* genes contribute about 15% of the total ethylene produced in *B. cinerea*-infected plants (Figure 3) [28]. Transcriptional activation of *ACS7*, *ACS8*, and *ACS11* is not regulated by the MPK3/MPK6 cascade (Figure 4); the signaling pathway(s) involved in the activation of their expression is unknown. It is also unclear whether ACS7, ACS8, and ACS11 are regulated at the protein stability level. Since ACS7 and ACS11 do not have a typical putative phosphorylation site in their C-termini, they are likely to be regulated at the transcriptional level only. ACS8, similar to ACS5 and ACS9, has a putative CDPK phosphorylation site in its C-terminus. It is possible that phosphorylation by CDPK(s) is involved in its protein stability regulation, similar to ACS5 and ACS9 [32], [33].

Ethylene plays an important role in plant disease resistance. Using a high-order *acs* mutant, Tsuchisaka et al. (2009) demonstrated that ethylene production is essential to plant resistance against *B. cinerea*. However, ethylene induction was not examined in the study. In this report, we demonstrate that ethylene induction in *acs1-1/acs2-1/acs6-1/acs4-1/acs5-2/acs9-1/acs7-1/acs11-1* mutant plants after *B. cinerea* infection is only at ∼2% of that in the wild type (Figure 3). Plant sensing of abiotic stress stimuli or invading pathogens triggers a number of signaling events. Among them, the activation of MAPK cascades and calcium influx are two of the earliest [21], [24], [52]. Our research demonstrates the regulation of ACS2 and ACS6 by a specific MAPK cascade at both transcriptional and post-translational levels. This pathway contributes ∼85% of the total ethylene induction in plants challenged by pathogens. Regulation of the remaining ACS isoforms is unclear at present. Additional studies, including identification of the signaling pathway(s) involved in regulation of *ACS7*, *ACS8*, and *ACS11* expressions, protein phosphorylation, and protein stability, are needed to further our understanding of the complex regulation of ethylene induction during the plant stress/defense response.

## Materials and Methods

### Plant growth conditions and treatments

Soil-grown plants were maintained at 22°C in a growth chamber with a 14-hr light cycle (100 µE/m^−2^ sec^−1^). For experiments, seeds were surface-sterilized. After imbibition at 4°C for 3–5 days, seeds were sown in petri dishes with liquid half-strength Murashige and Skoog (MS) medium and grown in a growth chamber at 22°C with continuous light (70 µE/m^−2^ sec^−1^). Five-day-old seedlings were transferred to 20-ml GC vials with 6 ml of liquid half-strength MS medium (10 seedlings per vial) and maintained under the same growth conditions. Twelve- to fourteen-day-old seedlings grown in GC vials were used for experiments.

Procedures for *Botrytis cinerea* (Strain: DSM 4709) maintenance and spore preparation were described previously [28]. Twelve-day-old seedlings grown in GC vials were inoculated with *B. cinerea* spores at a final concentration of 4.0×10^5^ spores/vial. Induction of *DD* and *ACS6* expressions in *GVG-NtMEK2^DD^* and *GVG-ACS6* transgenic plants was performed by the addition of DEX stock solution (5 mM in ethanol) to a final concentration of 1 µM. An equal volume of ethanol was used as a negative (−DEX) control.

At least two independent repetitions were performed with similar results for experiments with multiple time points. For single time-point experiments, at least three independent repetitions were performed.

### Mutant lines and generation of transgenic plants

*Arabidopsis thaliana* Columbia (Col-0) ecotype was used as the wild-type control, unless stated otherwise. T-DNA insertion mutant alleles of *MPK3* (At3g45640), *MPK6* (At2g43790), *ACS1* (At3g61510), *ACS2* (At1g01480), and *ACS6* (At4g11280) were described previously [16], [28], [39]. The two *ACS7* (At4g26200) mutant alleles, *acs7-1* (FLAG_431D05) and *acs7-2* (CSHL_ET5768), were obtained from INRA and Cold Spring Harbor Laboratory, respectively. High-order *acs* mutants generated in Dr. Athanasios Theologis' laboratory [35] were obtained from the Arabidopsis Biological Resource Center (ABRC). The stock numbers are CS16564 (*acs2-1*), CS16569 (*acs6-1*), CS16581 (*acs2-1/acs6-1*), CS16603 (*acs2-1/acs6-1/acs4-1*), CS16607 (*acs2-1/acs6-1/acs5-2*), CS16609 (*acs2-1/acs6-1/acs9-1*), CS16644 (*acs2-1/acs6-1/acs4-1/acs5-2/acs9-1*), CS16649 (*acs2-1/acs6-1/acs4-1/acs5-2/acs9-1/acs7-1*), CS16650 (*acs1-1/acs2-1/acs6-1/acs4-1/acs5-2/acs9-1/acs7-1*), and CS16651 (*acs1-1/acs2-1/acs6-1/acs4-1/acs5-2/acs9-1/acs7-1/acs11-1*). Conditionally rescued *mpk3/mpk6* double mutant was generated by transformation of DEX-inducible promoter driven *MPK6* cDNA (*GVG-MPK6*) into *mpk3^−/−^/mpk6^+/−^* plants [39]. Double mutant seedlings were recovered from seeds of *mpk3^−/−^/mpk6^+/−^/GVG-MPK6* plants sprayed with 30 µM DEX during the flowering stage. *GVG-NtMEK2^DD^* (abbreviated as *DD*), *DD/mpk3*, and *DD/mpk6* lines were previously described [28], [38].

The DEX-inducible promoter driven *ACS6* construct (*GVG-ACS6*) was generated by cloning the *ACS6* ORF with a 4xmyc tag [29] into the Xho I/Spe I sites of the pTA7002 vector [53]. The binary vector was transformed into *Agrobacterium tumefaciens* strain GV3101. Arabidopsis transformation was performed by the floral dip procedure [54], and transformants were identified by screening for hygromycin resistance. Independent lines with *ACS6* transgene induction were identified based on real-time qPCR analysis.

### Ethylene measurement

GC vials with Arabidopsis seedlings were flushed and capped immediately after treatment. At indicated times, ethylene levels in the headspace of the GC vials were measured by gas chromatography as previously described [16]. Seedlings were then collected, weighed, frozen in liquid nitrogen, and stored at −80°C for future analysis.

### RNA extraction and real-time PCR analysis

Total RNA was extracted using the Trizol reagent (Invitrogen). After DNase treatment, RNA (2 µg) was used for reverse transcription. Real-time PCR analysis was performed using an Opticon™ 2 real-time PCR machine as described previously [38]. The transcript of the *EF1α* gene was used to normalize the samples. Relative gene expression was calculated using two different methods. The first method is the commonly used double ΔCt method, which gives fold of gene induction relative to basal level before treatment (0 hr time point). The second method expresses the transcript level relative to that of the *EF1α* gene in the same sample, which is a better method when comparison of expression levels of different genes is necessary. The primers used for real-time PCR were *ACS1* (At3g61510, 5′-ACGCTTTTCTCGTCCCTACTC-3′ and 5′-GGCCTTAAGGTACGCTGATTC-3′), *ACS2* (At1g01480, 5′-GGATGGTTTAGGATTTGCTTTG-3′ and 5′-GCACTCTTGTTCTGGATTACCTG-3′), *ACS4* (At2g22810, 5′-AACAACCTTGTGCTCACTGCT-3′ and 5′-AGATCCCTATCAAACCCTGGA-3′), *ACS5* (At5g65800, 5′-GACTCTCATGTTTTGCCTTGC-3′ and 5′-TTGGAAGCCATTAGAGCTTGA-3′), *ACS6* (At4g11280, 5′-GTTCCAACCCCTTATTATCC-3′ and 5′-CCGTAATCTTGAACCCATTA-3′), *ACS7* (At4g26200, 5′-ACGGTACGATACCATTGTGGA-3′ and 5′-GCTCGCCGTCTTTAGTTTTCT-3′), *ACS8* (At4g37770, 5′-CCTTCCTTCCTTCAAGAATGC-3′ and 5′-GAGAGTCTCGTTAGCCGGAGT-3′), *ACS9* (At3g49700, 5′-CATACCTCGACGAAAACCAGA-3′ and 5′-TCATGTCAACCCAACAGAACA-3′), *ACS11* (At4g08040, 5′-CAAACGATGGAGGTTGCTATG-3′ and 5′-TTGGAGACCCATTTGTTGATAAG-3′), and *EF1α* (At5g60390, 5′-TGAGCACGCTCTTCTTGCTTTCA-3′ and 5′-GGTGGTGGCATCCATCTTGTTACA-3′).

### ChIP–qPCR analysis

F1 plants generated from the cross of *wrky33/4myc-WRKY33* and *DD* lines were used for the ChIP assay. Two-week-old seedlings treated with 1-µM DEX for 12 hr were processed as previously described [55]. Briefly, chromatin was isolated from 0.8 g of frozen tissue and sonicated with a Bioruptor sonicator (15 s on and 15 s off cycles, medium-energy settings) for 6 min. Immunoprecipitation was performed by incubating chromatin with 2 µg of anti-myc antibody (Millipore) or mouse IgG (negative control) for 1 hr at 4°C. The protein-chromatin immunocomplex was captured using Protein G-Dynal magnetic beads (Invitrogen). After Proteinase K digestion, the immunoprecipitated DNA was purified using a ChIP DNA Clean and Concentrator kit (Zymo Research Corporation). Immunoprecipitated DNA and input DNA were analyzed by qPCR using primers specific for the promoter regions of *PAD3* and *WRKY33*. The primer pairs (forward and backward) used for ChIP-qPCR were *ACS2* (5′-AGGCCATAAGCCCATTCAAA-3′ and 5′-GCCTACAGTGCACGACTTCA-3′) and *ACS6* (5′-AAAGTCGTTGAGATTGTGTTGG-3′ and 5′-TGGCAGCCTTAAAGACCAGT-3′), which are in proximity of the W-boxes in the promoters. ChIP results are presented as percentage of input DNA.

### Accession numbers

Sequence data for this article can be found in the Arabidopsis Genome Initiative or GenBank/EMBL databases under the following accession numbers: *MPK3* (At3g45640), *MPK6* (At2g43790), *EF1α* (At5g60390), *ACS1* (At3g61510), *ACS2* (At1g01480), *ACS4* (At2g22810), *ACS5* (At5g65800), *ACS6* (At4g11280), *ACS7* (At4g26200), *ACS8* (At4g37770), *ACS9* (At3g49700), *ACS11* (At4g08040), and *WRKY33* (At2g38470).

## Supporting Information

Figure S1 Mutation in *ACS7*, a Type III ACS isoform, slightly reduced *B. cinerea*-induced ethylene production. Two-week-old *acs7-1* and *acs7-2* as well as their respective wild-type controls, Ws-0 and Ler-0, grown in GC vials were inoculated with *B. cinerea* spores. Ethylene accumulation in the headspace was determined at the indicated times. Error bars indicate standard deviations (n = 3).
(TIF)

Figure S2 Comparison of basal-level and *B. cinerea*-induced ethylene production in *acs2-1/acs6-2* and *acs2-1/acs6-1* double mutants. (A) *B. cinerea*-induced ethylene production in wild type, *acs2-1*/*acs6-2*, and *acs2-1/acs6-1* plants. Twelve-day-old seedlings grown in GC vials were inoculated with *B. cinerea* spores. Ethylene accumulation in GC vials was monitored at indicated times. Error bars indicate standard deviations (n = 3). (B) Basal level ethylene production in wild type, *acs2-1*/*acs6-2*, and *acs2-1/acs6-1* seedlings. Twelve-day-old seedlings grown in GC vials were mock inoculated. Ethylene accumulation in GC vials was measured after 24 hours. Error bars indicate standard deviations (n = 3).
(TIF)

Figure S3 Basal level ethylene production in various *acs* mutants. (A) Basal level ethylene production in wild type (Col-0, *acs2-1*/*acs6-2* double and *acs2-1/6-2/7-1* triple mutant. Twelve-day-old seedlings grown in GC vials were mock inoculated. Ethylene accumulation in GC vials was measured 24 hours later. Error bars indicate standard deviations (n = 3). (B) Basal level ethylene production in the high-order *acs* mutants generated in Dr. Athanasios Theologis' lab. Twelve-day-old seedlings grown in GC vials were mock inoculated. Ethylene accumulation in GC vials was measured 24 hours later. Error bars indicate standard deviations (n = 3). The allele numbers are omitted for easy labeling. They are *acs1-1*, *acs2-1*, *acs4-1*, *acs5-2*, *acs6-1*, *acs7-1*, *acs9-1*, and *acs11-1*.
(TIF)

Figure S4 Activation of *ACS7*, *ACS8*, and *ACS11* in the *wrky33* mutant after *B. cinerea* inoculation. *B. cinerea*-induced *ACS7*, *ACS8*, and *ACS11* expression is not compromised in *wrky33* mutant. Total RNA from the experiment shown in Figure 8 was reverse transcribed. Expressions of *ACS7* (A), *ACS8* (B), and *ACS11* (C) genes were quantified by real-time PCR. *ACS* transcript levels were calculated as percentage of *EF1α* transcript. Error bars indicate standard deviations (n = 3).
(TIF)

Figure S5 Ethylene production in *acs2-1/acs6-2/acs7-1/GVG-ACS6* transgenic seedlings after DEX treatment. Twelve-day-old wild-type (Col-0), *acs2-1/acs6-2/acs7-1*, and *acs2-1/acs6-2/acs7-1/GVG-ACS6* transgenic seedlings (line #5 and #12) grown in GC vials were treated with DEX (+DEX, final concentration of 1 µM) or ethanol solvent control (−DEX), but without *B. cinerea* inoculation. Ethylene accumulation in GC vials was measured after 24 hours. Error bars indicate standard deviations (n = 3).
(TIF)
