# Supplementary figures and images for: Dual-Level Regulation of ACC Synthase Activity by MPK3/MPK6 Cascade and Its Downstream WRKY Transcription Factor during Ethylene Induction in Arabidopsis
Source: PLoS Genet. 2012 Jun 28;8(6):e1002767. doi: 10.1371/journal.pgen.1002767 (PMC3386168; doi:10.1371/journal.pgen.1002767)

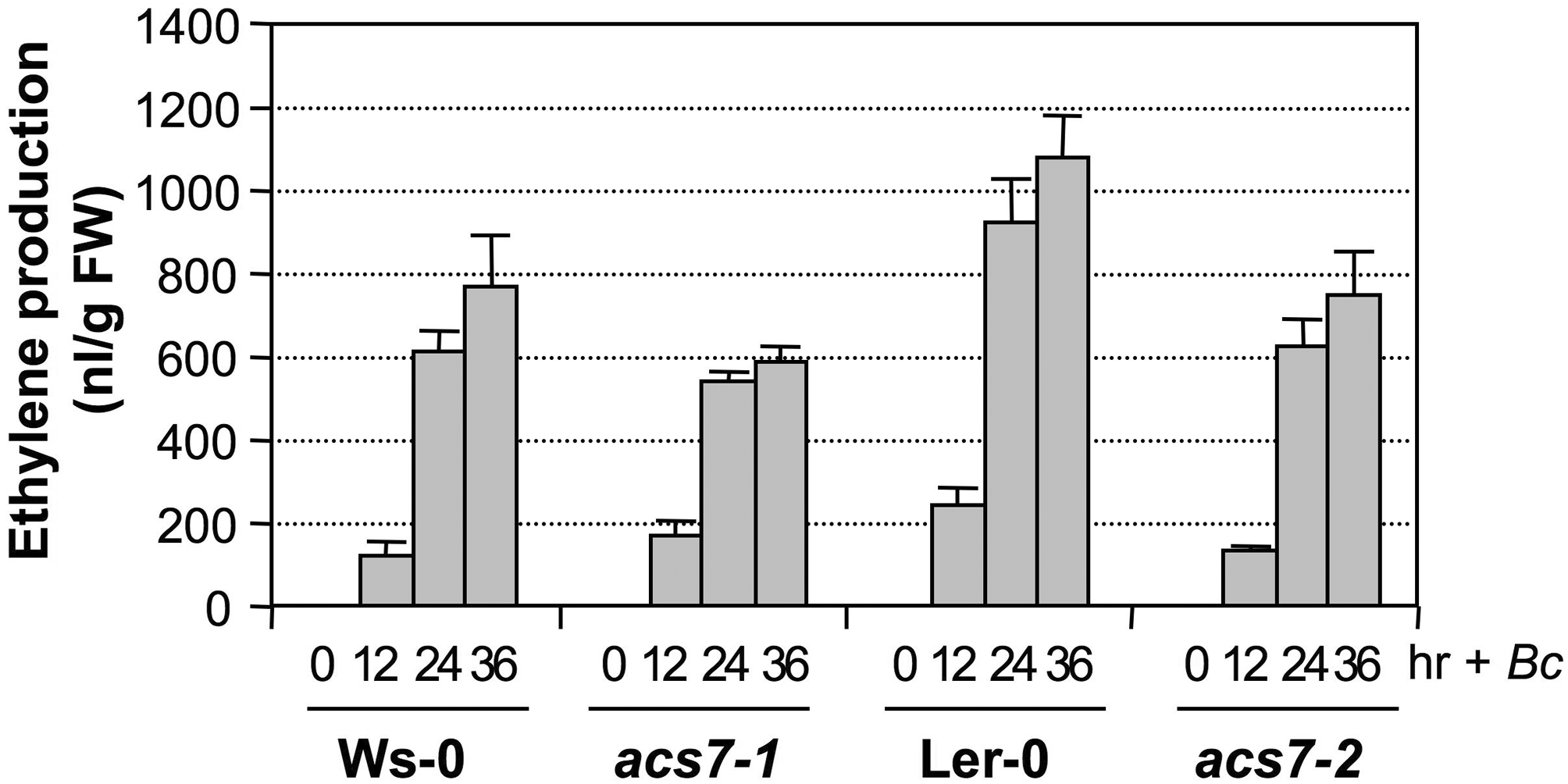

Supplement: Figure S1 — Mutation in ACS7, a Type III ACS isoform, slightly reduced B. cinerea-induced ethylene production. Two-week-old acs7-1 and acs7-2 as well as their respective wild-type controls, Ws-0 and Ler-0, grown in GC vials were inoculated with B. cinerea spores. Ethylene accumulation in the headspace was determined at the indicated times. Error bars indicate standard deviations (n = 3). (TIF) [file pgen.1002767.s001.tif]

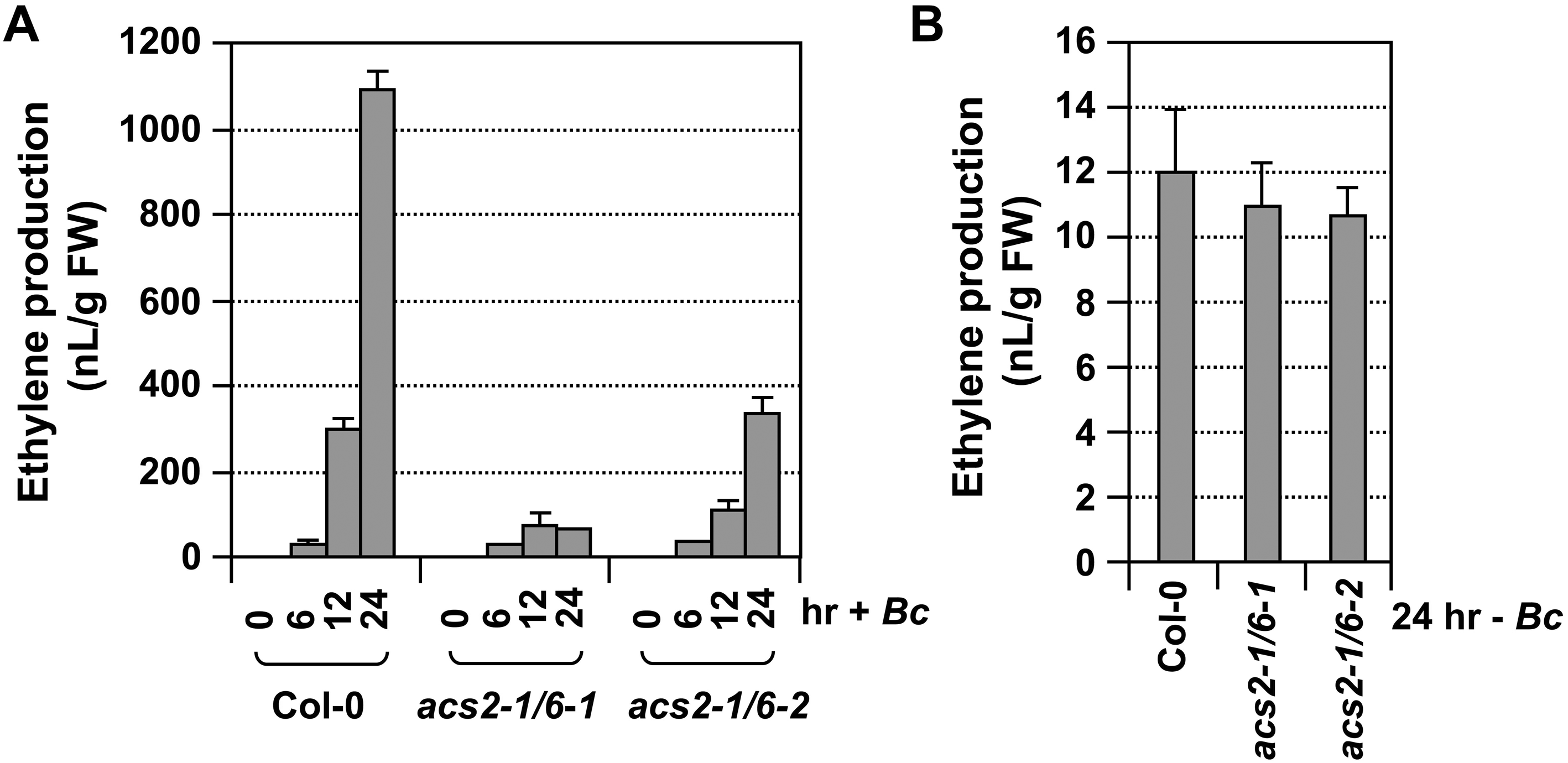

Supplement: Figure S2 — Comparison of basal-level and B. cinerea-induced ethylene production in acs2-1/acs6-2 and acs2-1/acs6-1 double mutants. (A) B. cinerea-induced ethylene production in wild type, acs2-1/acs6-2, and acs2-1/acs6-1 plants. Twelve-day-old seedlings grown in GC vials were inoculated with B. cinerea spores. Ethylene accumulation in GC vials was monitored at indicated times. Error bars indicate standard deviations (n = 3). (B) Basal level ethylene production in wild type, acs2-1/acs6-2, and acs2-1/acs6-1 seedlings. Twelve-day-old seedlings grown in GC vials were mock inoculated. Ethylene accumulation in GC vials was measured after 24 hours. Error bars indicate standard deviations (n = 3). (TIF) [file pgen.1002767.s002.tif]

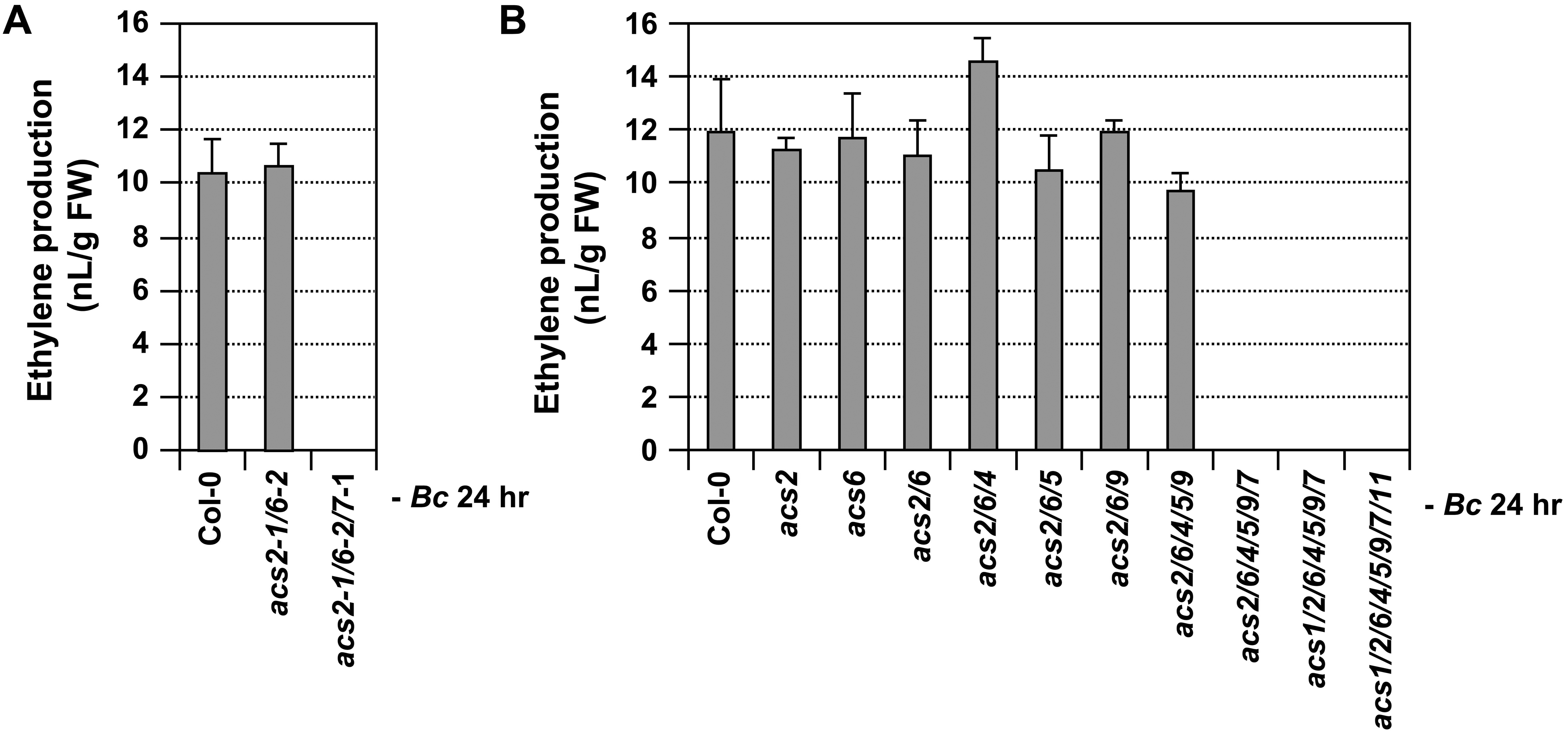

Supplement: Figure S3 — Basal level ethylene production in various acs mutants. (A) Basal level ethylene production in wild type (Col-0, acs2-1/acs6-2 double and acs2-1/6-2/7-1 triple mutant. Twelve-day-old seedlings grown in GC vials were mock inoculated. Ethylene accumulation in GC vials was measured 24 hours later. Error bars indicate standard deviations (n = 3). (B) Basal level ethylene production in the high-order acs mutants generated in Dr. Athanasios Theologis' lab. Twelve-day-old seedlings grown in GC vials were mock inoculated. Ethylene accumulation in GC vials was measured 24 hours later. Error bars indicate standard deviations (n = 3). The allele numbers are omitted for easy labeling. They are acs1-1, acs2-1, acs4-1, acs5-2, acs6-1, acs7-1, acs9-1, and acs11-1. (TIF) [file pgen.1002767.s003.tif]

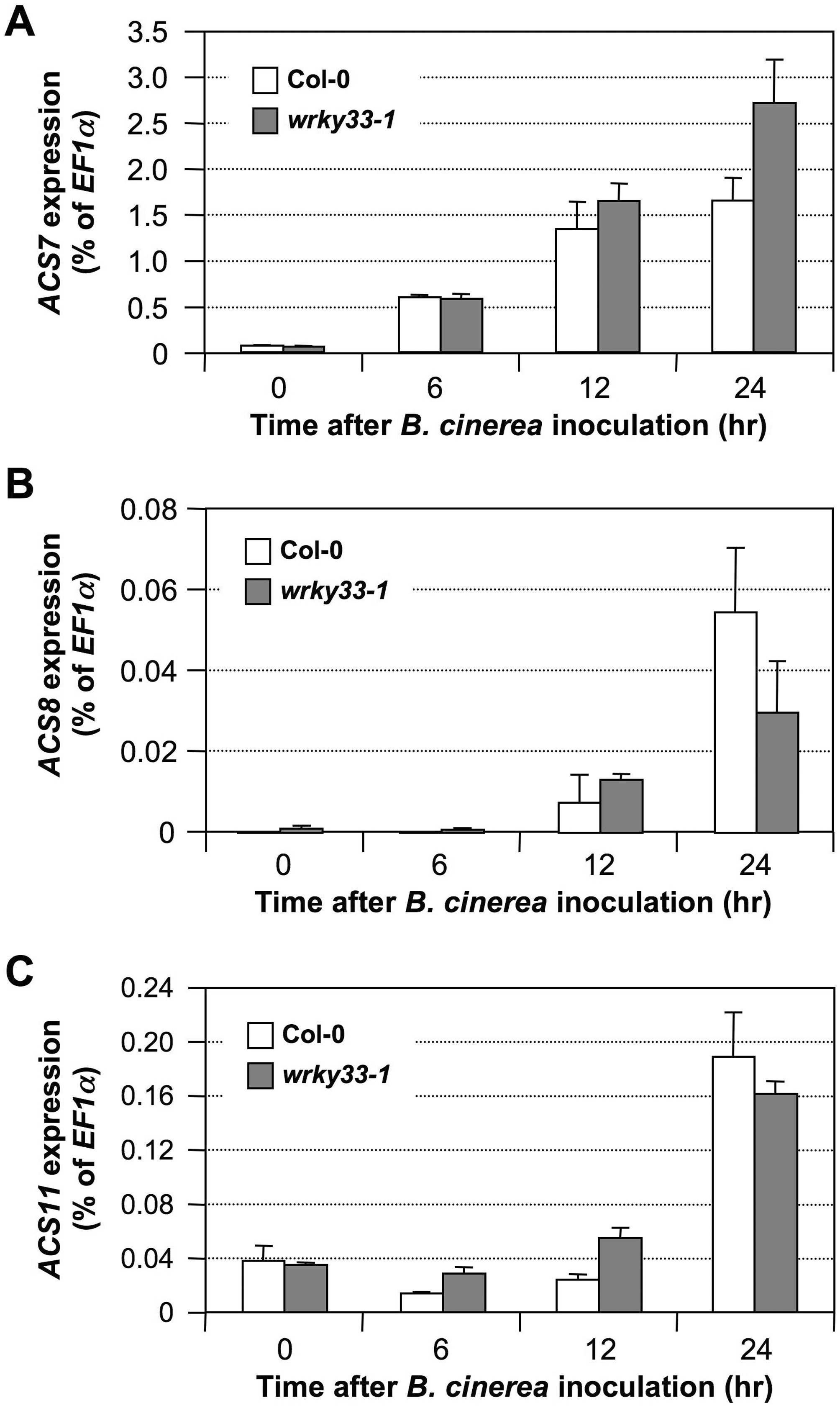

Supplement: Figure S4 — Activation of ACS7, ACS8, and ACS11 in the wrky33 mutant after B. cinerea inoculation. B. cinerea-induced ACS7, ACS8, and ACS11 expression is not compromised in wrky33 mutant. Total RNA from the experiment shown in Figure 8 was reverse transcribed. Expressions of ACS7 (A), ACS8 (B), and ACS11 (C) genes were quantified by real-time PCR. ACS transcript levels were calculated as percentage of EF1α transcript. Error bars indicate standard deviations (n = 3). (TIF) [file pgen.1002767.s004.tif]

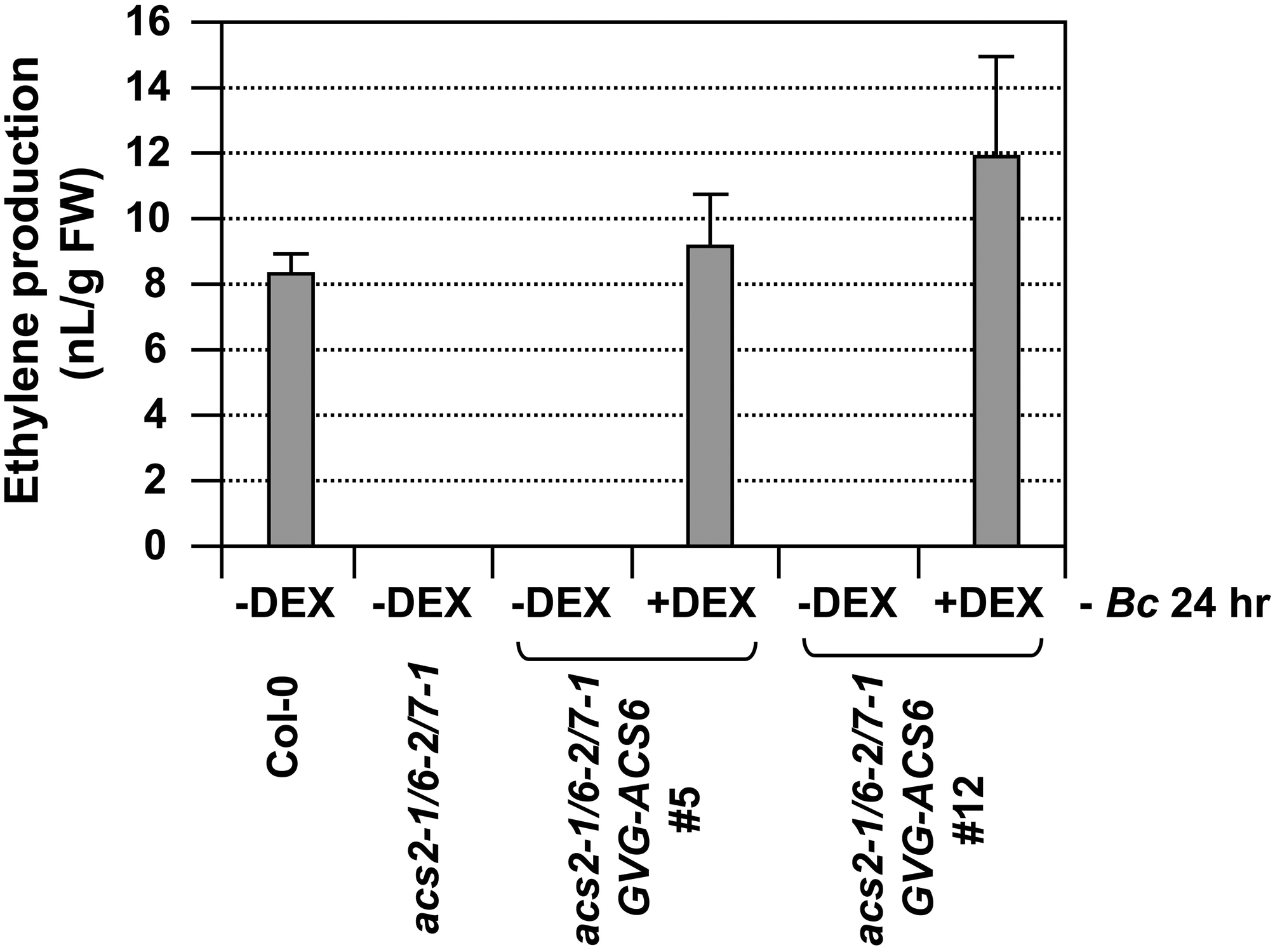

Supplement: Figure S5 — Ethylene production in acs2-1/acs6-2/acs7-1/GVG-ACS6 transgenic seedlings after DEX treatment. Twelve-day-old wild-type (Col-0), acs2-1/acs6-2/acs7-1, and acs2-1/acs6-2/acs7-1/GVG-ACS6 transgenic seedlings (line #5 and #12) grown in GC vials were treated with DEX (+DEX, final concentration of 1 µM) or ethanol solvent control (−DEX), but without B. cinerea inoculation. Ethylene accumulation in GC vials was measured after 24 hours. Error bars indicate standard deviations (n = 3). (TIF) [file pgen.1002767.s005.tif]
